# Supplementary material for: The VIPR-1 trial (Visualizing Ischemia in the Pancreatic Remnant): Assessing the role of intraoperative indocyanine green perfusion in predicting postoperative pancreatic leaks and fistulas: Protocol for a phase II clinical trial
Source: PLoS One. 2025 Jun 24;20(6):e0311025. doi: 10.1371/journal.pone.0311025 (PMC12186879; doi:10.1371/journal.pone.0311025)
Supplement: S2 File — (PDF) [file pone.0311025.s002.pdf]

KNIGHT CANCER Institute
OHSU

MEMORANDUM

DATE: September 6, 2023

TO: Patrick Worth, MD

FROM: Stephen Spurgeon, MD
Skye Mayo, MD, MPH
Clinical Research and Review Committee (CRRC) Chairs
OHSU Knight Cancer Institute

RE: IRB 25055: Assessing the Role of Intraoperative Indocyanine Green Perfusion of the Transected Pancreas in Predicting Postoperative Pancreatic Leaks

---

Administrative Review Category:
Non-Interventional Trial (ie Retrospective, Observational, Ancillary/Correlative)

CRRC Determination:
The Knight Clinical Research Review Committee (CRRC) conducted an Administrative Review of the above mentioned study on August 21, 2023.

Upon receipt and review of the responses provided from the initial review, the CRRC administratively approved the study on September 6, 2023.

eCRIS Requirements:
All cancer-related human subject research, regardless of funding or IRB of record, requires an eCRIS submission. Study-level data must be entered in eCRIS at study startup & maintained throughout the trial. Depending on the type of study, certain subject-level data (either full or limited) must also be entered & maintained in eCRIS.

The study requires the following level of reporting:

	REPORTING LEVEL	REPORTING REQUIREMENTS
☒	Study-Level Data and **Full** Subject-Level Data	Subject Level: Refer to Knight eCRIS SOP CR016 for specific required fields.
☐	Study-Level Data and **Limited** Subject-Level Data	
☐	Study-Level Data Only	Study Level: General Information Smartform,

Knight requires all *study-level* information to be entered and maintained in eCRIS. Use of other system functionality (e.g. visit schedule or budget) is determined by institution requirements or clinical research group determination. Once the study is activated in eCRIS, the Knight requires that study-level information be updated as needed. For step by step instructions on the Knight requirements including updating subject level data, reference the eCRIS toolkit on the CRM Sharepoint page. For general eCRIS instructions on creating an eCRIS submission, consult the eCRIS user manual maintained by the eCRIS trainer. For affiliate sites, consult with appropriate IRB to ensure study authorizations are accurate and IRB approved prior to releasing PHI to the Knight Cancer Institute.

Ongoing CRRC Requirements:
All future protocol revisions or amendments and continuing reviews must be reviewed by the OHSU Knight Cancer Institute prior to IRB approval. All eIRB modifications, reportable events (i.e. unanticipated problems, protocol deviations) and continuing review submissions for this study will be automatically routed through the OHSU Knight Cancer Institute for review.

If you have questions about the CRRC determination or review process, please refer to the Knight CRRC Bridge page or contact knightcrrc@ohsu.edu

PRMS Reliance Information: The OHSU Knight Cancer Institute's Protocol Review and Monitoring System (PRMS) received a conditional approval from the National Cancer Institute (NCI) effective July 1, 2022. Per NCI regulations, no other NCI-designated cancer center should rely on our PRMS review until granted full approval.
