# Supplementary material for: The VIPR-1 trial (Visualizing Ischemia in the Pancreatic Remnant): Assessing the role of intraoperative indocyanine green perfusion in predicting postoperative pancreatic leaks and fistulas: Protocol for a phase II clinical trial
Source: PLoS One. 2025 Jun 24;20(6):e0311025. doi: 10.1371/journal.pone.0311025 (PMC12186879; doi:10.1371/journal.pone.0311025)
Supplement: S3 File — (DOCX) [file pone.0311025.s003.docx]

| **Full Study Title:** | Assessing the Role of Intraoperative Indocyanine Green Perfusion of the Transected Pancreas in Predicting Postoperative Pancreatic Leaks |
| --- | --- |
| **Protocol Number:** | IRB00025055 |
| **Investigational Product:** | indocyanine green |
| Version Number: | 1.1 |
| Version Date: | 10-17-23 |
| Replaces: | n/a |
| Principal Investigator: | Patrick J. Worth, MD, FACS, FSSO |
|  | Surgery  Oregon Health & Science University  2730 SW Moody Ave, CL4RS  Portland, OR 97239  Phone: 503-346-0243  Fax: n/a  Email: worth@ohsu.edu |
| **Regulatory Sponsor:**  **(if different)** | **n/a** |
| **Statistician:** | Jeong Youn Lim, PhD  Biostatistics Shared Resource  Phone: 503 418-0127  Fax:  Email: limje@ohsu.edu |

| # | **Section** | **Summary of Changes** | **Justification** |
| --- | --- | --- | --- |
| 1. |  |  |  |
| 2. |  |  |  |
| 3. |  |  |  |
| 4. |  |  |  |
| 5. |  |  |  |

| **Full Study Title** | Assessing the Role of Intraoperative Indocyanine Green Perfusion of the Transected Pancreas in Predicting Postoperative Pancreatic Leaks |
| --- | --- |
| **Protocol #** | IRB00025055 |
| **Coordinating Center** | OHSU, Knight Cancer Institute |
| **Clinical Phase** | other (specify) – Phase II Application of Approved Device and Drug |
|  | |
| **Study Description** | This study is designed to evaluate the association between intraoperative perfusion of the remnant pancreas and postoperative pancreatic fistula (leak) rates. Using an FDA approved drug (indocyanine green) and device (Stryker Spy Elite) for near-infrared imaging, we will assess perfusion of the remnant pancreas for patients undergoing pancreatectomy. We will then follow their postoperative course to evaluate whether a pancreatic leak developed, to determine if intraoperative perfusion correlates with leak rates. The aim of this study is to collect preliminary data on feasibility and association, with the ultimate goal of determining whether future investigation into modifying pancreatic perfusion will decrease rates of pancreatic leak. |
|  | |
| **Primary Objective** | Evaluate association between postoperative pancreatic fistula development and intraoperative pancreatic remnant perfusion. |
| **Secondary Objectives** | Evaluate the grade of leak via ISGPS classification and ICG metrics  Determine feasibility of intraoperative measurement of pancreatic perfusion using ICG and the SpyElite platform via a survey to be completed by OR staff.  Evaluate logistics of intraoperative perfusion measurement for staff and operating room using a survey. |
| **Primary Endpoint** | Incidence of biochemical leak or fistula after pancreatectomy defined by definitions of the ISGPS criteria |
|  | |
| **Key Inclusion Criteria** | Patients undergoing open pancreaticoduodenectomy or distal pancreatectomy for any diagnosis will be evaluated for inclusion. |
| **Key Exclusion Criteria** | Development of intra- or post-operative occurrences known to be associated with postoperative pancreatic fistula including hemorrhage, intraoperative blood transfusion, sustained postoperative hypotension requiring vasopressor support, unexpected ICU transfer, initiation of ACLS protocols will be excluded.  Patients with adverse reactions or allergies to indocyanine green will be excluded. History of prior pancreatectomy, prior severe pancreatitis, history of chronic pancreatitits, visceral vascular abnormalities, severe atherosclerosis, planned vascular reconstruction, hepatic insufficiency, liver fibrosis or cirrhosis will be excluded. |
| **Number of Participants** | *n* = 50 patients; anticipate screening *n =* 75 individuals. |
| **Duration of Therapy** | One 5.0 mg IV dose of indocyanine green, intraoperatively, with repeat intraoperative dosing up to maximum 2mg/kg |
| **Duration of Follow Up** | Thirty (30) days. |
|  | |
| **Description of Study Intervention** | Study participants will undergo one intraoperative measurement of pancreatic remnant perfusion. This involves intravenous administration of indocyanine green dye during their operation followed by immediate near-infrared perfusion measurement using the Stryker SpyElite camera, targeted at the organ of interest prior to drug administration. The intervention is expected to take five minutes total. |
|  | |
| **Statistical Analyses** | Chi-square test will be used to evaluate the association between leaks and perfusion. Logistic regression analysis will be further utilized to evaluate covariates influencing the presence/absence of leaks, with specific attention to the predictive value of percent maximum perfusion metrics and time to maximum metrics at the set timepoints in the protocol. With leaks of any severity occurring in a minimum 20% of pancreatectomies based on extensive published data. Our projected sample size of 50 will achieve 80% power to detect at least 25% difference in leaks rate between two group assuming 20% leak rate for normal ICG perfusion group.  We plan to enroll patients on a rolling basis. With a liberal estimated exclusion of 30% due to the multiple and complex postoperative factors known to be associated with leak (hemorrhage, sustained hypotension, vasopressor requirements included in “Exclusion Criteria”) we anticipate screening 75 patients and enrolling 50. The overall study will be paused if the following events occur: Life-threatening grade 4 toxicity attributable to protocol therapy that is unmanageable, or unexpected, death suspected to be related to ICG injection, as indicated by statistical stopping rules in this protocol. |

SCHEMATIC OF STUDY DESIGN


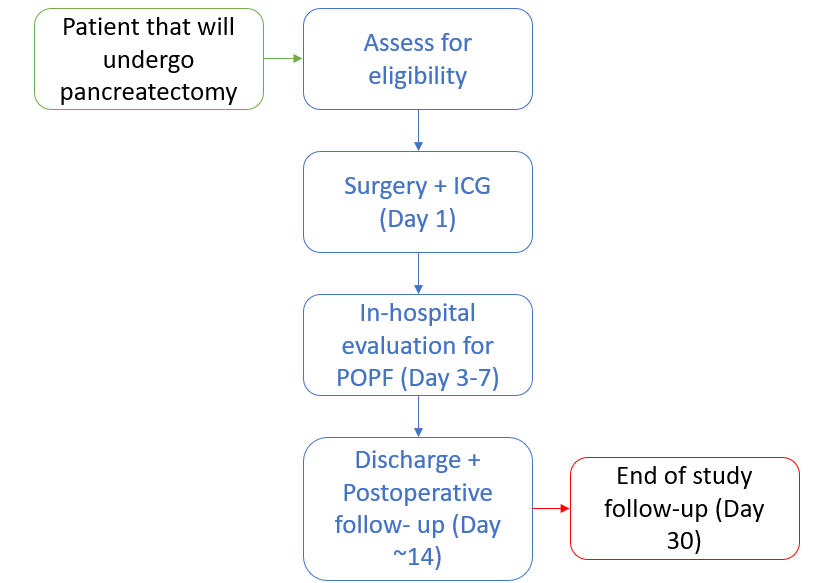


|  | **Preop visit** | **Surgery and Postoperative Period** | | | | |
| --- | --- | --- | --- | --- | --- | --- |
| **Procedure** |  | **Day 1 (Day of Surgery)** | **Day 4±3**  **(Post-operative Day 3)** | **Day of Discharge** | **Discharge + 7-14 Days (Post-Operative Follow-up)** | **Day 30** |
| Physical exam | x | X | X | X | X |  |
| Vital signs^1^ | x | X | X | X | X |  |
| ECG (if indicated) | x | X |  |  |  |  |
| Routine blood draw^2^  (1 tablespoon) |  | X | X |  |  |  |
| Drain Fluid Analysis for amylase |  |  | X |  |  |  |
| Pregnancy test (if indicated) | x | X |  |  |  |  |
| Review any adverse events |  | X |  |  | X | X |
| Review all other medications |  | X |  |  |  |  |
| ICG |  | X |  |  |  |  |
| Pancreas Resection |  | X |  |  |  |  |

TABLE OF CONTENTS

[TABLE OF CONTENTS 7](#_Toc1924134513)

[LIST OF TABLES 7](#_Toc453567276)

[LIST OF FIGURES 7](#_Toc1478094859)

[LIST OF ABBREVIATIONS 7](#_Toc1089401493)

[0. BACKGROUND INFORMATION AND SCIENTIFIC RATIONALE 9](#_Toc2112760570)

[0.0 Background 9](#_Toc1747993032)

[0.0.0 Overview of study disease(s) 9](#_Toc2072564894)

[0.0.1 Overview study intervention(s) 10](#_Toc2069882099)

[0.1 Study Rationale 11](#_Toc1540543954)

[0.1.0 Known Potential Risks 11](#_Toc255108729)

[0.1.1 Known Potential Benefits 11](#_Toc741228814)

[1. OBJECTIVES AND ENDPOINTS 11](#_Toc1356445596)

[1.0 Primary Objective and Endpoint 11](#_Toc447458419)

[1.1 Secondary Objectives and Endpoints 12](#_Toc2126476273)

[2. STUDY DESIGN 12](#_Toc1152750524)

[2.0 Description of the Study Design 12](#_Toc1870469471)

[2.1 End of Study Definition 13](#_Toc283376509)

[2.2 Study Discontinuation and Closure 13](#_Toc2009768582)

[3. STUDY POPULATION 13](#_Toc2134264063)

[3.0 Participant Inclusion Criteria 14](#_Toc1583106511)

[3.1 Participant Exclusion Criteria 14](#_Toc1557060107)

[3.2 Lifestyle Considerations 14](#_Toc750808681)

[3.3 Strategies for Recruitment and Retention 14](#_Toc237837087)

[3.3.0 Accrual Estimates 15](#_Toc1214115190)

[3.3.1 Inclusion of Children 16](#_Toc5492008)

[4. PARTICIPANT SCREENING, ENROLLMENT, AND WITHDRAWAL 16](#_Toc1576687643)

[4.0 Consent and Screening 16](#_Toc1263756479)

[4.0.0 Screening Period 17](#_Toc332532773)

[4.0.1 Re-testing During Screening 17](#_Toc119060762)

[4.1 Enrollment Procedures 17](#_Toc1641450276)

[4.1.0 Enrollment Procedures for the OHSU Site 17](#_Toc987010457)

[4.2 Measures to Minimize Bias 17](#_Toc1668591019)

[4.2.0 Randomization/Masking Procedures 18](#_Toc1985912375)

[4.2.1 Evaluation of Success of Blinding 18](#_Toc259509808)

[4.2.2 Breaking the Study Blind/Participant Code 18](#_Toc1528014018)

[4.3 Screen Failures 18](#_Toc1154545362)

[4.3.0 Re-Screening Allowance / Procedures 18](#_Toc190857462)

[4.4 Participant Discontinuation or Withdrawal 18](#_Toc1050660732)

[4.4.0 Handling Participant Discontinuation from Study 19](#_Toc1020193132)

[4.5 Lost to Follow-up 20](#_Toc1805875896)

[5. STUDY INTERVENTION 20](#_Toc1829491999)

[5.0 Name of Study Intervention 20](#_Toc818837081)

[5.0.0 Study Intervention Description 20](#_Toc5575535)

[5.0.1 Acquisition 20](#_Toc334383864)

[5.0.2 Formulation, Appearance, Packaging and Labeling 21](#_Toc1813309486)

[5.0.3 Product Storage and Stability 21](#_Toc905348854)

[5.0.4 Compatibility 21](#_Toc786970044)

[5.0.5 Handling 21](#_Toc1863325929)

[5.0.6 Preparation 21](#_Toc9345718)

[5.0.7 Administration 21](#_Toc765758815)

[5.0.8 Special Considerations for Administration 21](#_Toc115922598)

[5.0.9 Accountability 22](#_Toc513053564)

[5.1 Device Specific Considerations 23](#_Toc1162246053)

[6. TREATMENT PLAN 23](#_Toc1305374650)

[6.0 Dosage and Administration 23](#_Toc1874028087)

[6.1 Concomitant Medication and Supportive Care Guidelines 23](#_Toc1180713262)

[6.2 Precautionary Medications, Treatments, and Procedures 24](#_Toc889964556)

[6.3 Prohibited Medications, Treatments, and Procedures 24](#_Toc1917548347)

[7. STUDY PROCEDURES/EVALUATIONS AND SCHEDULE 24](#_Toc600010845)

[7.0 Study-Specific Procedures 24](#_Toc86635459)

[7.0.0 Medical history 24](#_Toc1096931310)

[7.0.1 Disease Assessment 24](#_Toc2061397353)

[7.0.2 Medication review 24](#_Toc1645107561)

[7.0.3 Physical examination 24](#_Toc408491364)

[7.0.4 Radiographic or other imaging assessments 25](#_Toc1176288817)

[7.0.5 Adverse event evaluation 25](#_Toc1253547827)

[7.0.6 Counseling procedures 25](#_Toc1507543915)

[7.0.7 Assessment of participant-reported outcomes 25](#_Toc1233284810)

[7.1 Laboratory Procedures and Evaluations 26](#_Toc628845313)

[7.2 Biomarker, Correlative, and Special Studies 26](#_Toc311997624)

[7.3 Screening Assessments 26](#_Toc712075375)

[7.4 Baseline Assessments 27](#_Toc1853998068)

[7.5 Early Termination or End of Treatment Visit 27](#_Toc1141331067)

[7.6 Follow-up 27](#_Toc958872613)

[7.7 Unscheduled Visits 27](#_Toc494547418)

[7.8 Schedule of Events 27](#_Toc1997616414)

[8. EFFICACY MEASURES 29](#_Toc1518740197)

[8.0 Definition of Efficacy Measures 30](#_Toc1682875834)

[8.1 Disease Evaluation 30](#_Toc2074985423)

[8.2 ISGPS DEFINITION AND GRADING OF POSTOPERATIVE PANCREATIC FISTULA 30](#_Toc1687857832)

[9. SAFETY 30](#_Toc252480616)

[9.0 Specification Of Safety Parameters 30](#_Toc2012483781)

[9.1 Definitions 30](#_Toc830028076)

[9.1.0 Adverse Event (AE) 30](#_Toc2107175400)

[9.1.1 Serious Adverse Event (SAE) 31](#_Toc484614986)

[9.1.2 Unanticipated Problems (UP) 31](#_Toc1258993470)

[9.1.3 Severity of Event 31](#_Toc1989840728)

[9.1.4 Assessment of Causality Relationship To Study intervention 32](#_Toc2020289839)

[9.2 Expectedness 32](#_Toc399865700)

[9.3 Adverse Event List(s) 32](#_Toc268496283)

[9.3.0 Adverse Event List for ICG INJECTION 32](#_Toc1350497252)

[9.4 Time Period And Frequency For Event Assessment And Follow-Up 32](#_Toc1176896709)

[9.5 Reporting Procedures 33](#_Toc1919747643)

[9.5.0 OHSU IRB Reporting of Unanticipated Problems and Adverse Events 33](#_Toc1696115972)

[9.5.1 Central Reporting of Adverse Events for Multi-Site Studies 33](#_Toc413122843)

[9.5.2 FDA Reporting 34](#_Toc1539185702)

[9.5.3 Suspected Unexpected Adverse Reactions (SUSARs) 34](#_Toc1009594292)

[9.5.4 Reporting Of Pregnancy 35](#_Toc474170285)

[9.6 Study Stopping Rules 35](#_Toc1015386055)

[10. STATISTICAL CONSIDERATIONS 35](#_Toc346992907)

[10.0 Statistical Hypothesis 35](#_Toc422752677)

[10.1 Sample Size Determination 35](#_Toc96112860)

[10.2 Populations for Analyses 36](#_Toc1832282531)

[10.3 Description of Statistical Methods 36](#_Toc347190633)

[10.3.0 General Approach 36](#_Toc2086096253)

[10.3.1 Analysis of Primary Endpoint(s) 36](#_Toc747076626)

[10.3.2 Analysis Of The Secondary Endpoint(S) 36](#_Toc1137525985)

[10.3.3 Safety Analyses 36](#_Toc226942395)

[10.3.4 Baseline Descriptive Statistics 37](#_Toc969931310)

[10.4 Handling of Missing Data 37](#_Toc958508524)

[11. CLINICAL MONITORING 37](#_Toc1584844645)

[11.0 OHSU Knight Cancer Institute Data & Safety Monitoring Plan 37](#_Toc1283357771)

[11.1 Clinical Data & Safety Monitoring 37](#_Toc174839870)

[11.2 Quality Assurance & Quality Control 38](#_Toc2079466550)

[12. SUPPORTING DOCUMENTATION AND OPERATIONAL CONSIDERATIONS 39](#_Toc501247283)

[12.0 Source Data/Documents 39](#_Toc1485493529)

[12.0.0 Participant & Data Confidentiality 39](#_Toc1856961242)

[12.0.1 Data Collection & Storage: Privacy, Confidentiality & Security 40](#_Toc1378787074)

[12.0.2 Future Use of Stored Specimens and Data 41](#_Toc79332220)

[12.0.3 Maintenance of Records 41](#_Toc1679892799)

[12.1 Multi-Site Guidelines 41](#_Toc47465201)

[12.2 Publication and Data Sharing Policy 41](#_Toc764428722)

[12.3 Conflict of Interest Policy 42](#_Toc764022328)

[13. ETHICS/PROTECTION OF HUMAN PARTICIPANTS 42](#_Toc1926648031)

[13.0 Ethical Standard 42](#_Toc41365790)

[13.1 Institutional Review Board 42](#_Toc902576127)

[13.2 Informed Consent 43](#_Toc1542480725)

[13.2.0 Consent Procedures and Documentation 43](#_Toc789283214)

[13.3 Protocol Review 43](#_Toc167616178)

[13.4 Changes to Protocol 43](#_Toc1195756763)

[14. REFERENCES 44](#_Toc646025231)

LIST OF TABLES

Right click on text below and select Update Field

[**Table 1.** **Population Demographics - Oregon (%)** 22](#_Toc135817272)

[**Table 2. Projected Accrual for the Present Study** 23](#_Toc135817273)

LIST OF FIGURES

Right click on text below and select Update Field

**No table of figures entries found.**

LIST OF ABBREVIATIONS

| AE | Adverse event |
| --- | --- |
| ALP | Alkaline phosphatase |
| ALT | Alanine aminotransferase |
| ANC | Absolute neutrophil count |
| AST | Aspartate aminotransferase |
| ATC | Anatomical Therapeutic Chemical (Classification System) |
| AUC | Area under the curve |
| BMA, BMB | Bone marrow aspiration/biopsy |
| BUN | Blood urea nitrogen |
| CBC | Complete blood cell (count) |
| CFR | United States Code of Federal Regulations |
| CoC | National Institutes of Health (NIH) Certificate of Confidentiality |
| CR | Complete response |
| CRC | Clinical Research Coordinator |
| CRMS | Clinical research management system |
| CRQA | Clinical Research Quality & Administration |
| CRRC | Clinical Research Review Committee (OHSU) |
| CRF | Case report form |
| CSF | Cerebral spinal fluid |
| CT | Computerized tomography |
| CTCAE | Common Terminology Criteria for Adverse Events |
| CTEP | Cancer Therapy Evaluation Program |
| CTMS | Clinical Trial Management System |
| DFS | Disease-free survival |
| DLT | Dose limiting toxicity |
| DP | Distal Pancreatectomy (cf. RAMPS) |
| DSMB | Data and Safety Monitoring Board |
| DSMC | Data and Safety Monitoring Committee |
| DSMP | Data and Safety Monitoring Plan |
| ECG, EKG | Electrocardiogram |
| ECOG | Eastern Cooperative Oncology Group |
| eCRF | Electronic Case Report Form |
| eCRIS | Electronic Clinical Research Information System |
| EDC | Electronic data capture |
| FCBP | Female of childbearing potential |
| FDA | United States Food and Drug Administration |
| GCP | Good Clinical Practice |
| HBeAg | Hepatitis B “e” antigen |
| HBV | Hepatitis B virus |
| HCT | Hematocrit |
| HCV | Hepatitis C virus |
| HGB | Hemoglobin |
| HIPPA | Health Insurance Portability and Accountability Act |
| HIV | Human immunodeficiency virus |
| IB | Investigator’s Brochure |
| ICF | Informed Consent Form |
| ICG | Indocyanine Green |
| ICH | International Conference on Harmonization |
| IDE | Investigational Device Exemption |
| IEC | Independent Ethics Committee |
| IND | Investigational new drug application |
| IP | Investigational product |
| IRB | Institutional Review Board |
| iwCLL | International Workshop on Chronic Lymphocytic Leukemia |
| IV | Intravenous |
| LDH | Lactate dehydrogenase |
| LFT | Liver function test |
| MedDRA | Medical Dictionary for Regulatory Activities |
| MRI | Magnetic resonance imaging |
| MTD | Maximum tolerated dose |
| MUGA | Multiple Gated Acquisition |
| N/A | Not applicable |
| NCI | National Cancer Institute |
| NHL | Non-Hodgkin’s lymphoma |
| OHRP | Office for Human Research Protections |
| OHSU | Oregon Health & Science University |
| ORR | Overall response rate |
| PD | Pancreaticoduodenoectomy (aka: Whipple Procedure) |
| PDAc | Pancreatic Ductal Adenocarcinoma (aka: Pancreatic Cancer) |
| PET | Positron emission tomography |
| PI | Principal Investigator |
| PK | Pharmacokinetics |
| PO | *Per os* (by mouth, orally) |
| POPF | Post-Operative Pancreatic Fistula (aka: “leak” or “fistula”) |
| PR | Partial response |
| QOL | Quality of Life |
| RAMPS | Radical anterograde modular pancreaticosplenectomy (Radical distal pancreatectomy) |
| RBC | Red blood cell (count) |
| RP2D | Recommended Phase II Dose |
| RNI | Reportable new information |
| RT | Radiation therapy |
| SAE | Serious adverse event |
| SD | Stable disease |
| SD | Standard deviation |
| SGOT | Serum glutamic oxaloacetic transaminase |
| SGPT | Serum glutamic pyruvic transaminase |
| TSMP | Trial Specific Monitoring Plan |
| UA | Urinalysis |
| ULN | Upper limit of normal |
| UP | Unanticipated problem |
| VAPORHCS | Veteran Affairs (VA) Portland Health Care System |
| WBC | White blood cell (count) |

# BACKGROUND INFORMATION AND SCIENTIFIC RATIONALE

## Background


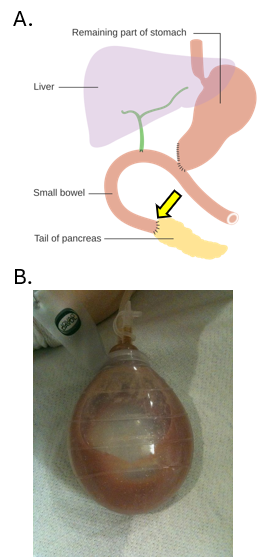


Cancer of the pancreas will cause more than 50,000 deaths this 2023 in the US alone. The incidence of pancreatic cancer has been growing by 1% a year for the last two decades.^1^Pancreatic cancer has one of the lowest overall 5-year survival rates of all organ tumors, with 5-year-survival rates estimated at around 10%. 5-year survival rates can increase to more than 30% when tumor and patient characteristics allow for resection.^2^ Open pancreaticoduodenectomy and distal pancreatectomy are the treatment of choice when tumors are limited to the pancreas. These surgical procedures have high rates of morbidity, with the most serious complication being pancreatic leaks. It is estimated that up to 30% of all patients that undergo removal of the pancreas develop a pancreatic leak. Typically, undetectable at time of surgery, pancreatic leaks occur at the pancreas to bowel anastomosis (fig 1a, yellow arrow). Diagnosis is done when clinical suspicion is high and entails measurement and analysis of suction drains (fig 1b). Pancreatic leaks prolong postoperative hospital length of stay, delay initiation of chemotherapy and increase risk for infection.^3^ Because adequate recovery is hindered by pancreatic leaks, minimizing the risk for this complication is of uttermost importance for improving patient outcomes. Recent studies have analyzed different methods to predict the risk for developing a pancreatic leak after pancreatectomy with variable success. However, these methods involve postoperative measurements. ^4, 5^ Because it arises from dual embryologic sources, the surgical neck of the pancreas is a poorly perfused watershed region of the gland, subject to hypoperfusion.^6, 7^ Ischemia of the pancreatic remnant is a factor for the development of leaks.^8-10^ Currently, surgeons rely on direct visualization of pancreatic tissue and ultrasound to estimate optimal blood flow. Present methods have been of limiting value in predicting adequate blood flow in both open pancreaticoduodenectomy and distal pancreatectomy.^11-12^ An improved intraoperative technique to analyze blood perfusion could guide surgical margins to minimize risk for pancreatic leaks. Indocyanine green (ICG) fluorescence is a method widely used to assess blood perfusion of tissues during surgery. ICG use is described in recent literature of pancreatectomy.^13-15^ However, there is no prospective systematic analysis of its use in assessing the risk of developing a pancreatic leak.

Figure 1. A) Reconstructed Whipple with typical leak site Image by Cancer Research UK / Wikimedia Commons; B) Closed suction drain. Public Domain Image. Images from Wikimedia Commons under Creative Commons Attribution (CC BY) 4.0 license.

- - 1. Overview of study disease(s)

**Pancreatic tumors**:

90% of all tumors of the pancreas are of the ductal adenocarcinoma type. With more uncommon tumors including neuroendocrine tumors, among others. While there are no clear-cut causes in most cases, patient risk factors include tobacco and alcohol use Genetic mutations with BRCA and ATM are considered known genetic patterns that increase risk for pancreatic adenocarcinoma. Up to half of all adenocarcinomas of the pancreas present symptoms once they are in a metastatic stage. Only about 1 in 5 patients are diagnosed in an early enough stage of disease where it is considered resectable by surgery.^16^

- - 1. Overview study intervention(s)

While the study is designed to examine the utility of near-infrared imaging and post-transection perfusion of the remnant pancreas on the prevalence of pancreatic leaks, ***the intervention planned is solely the addition of ICG administration and intraoperative near-infrared imaging***. Standard-of-care surgeries as planned by the attending physician are described below (“Distal pancreatectomy” and “Open pancreaticoduodenectomy”) **but are not considered study interventions.**

**Distal pancreatectomy**:

Encompasses the removal of the last part of the pancreas and sometimes removing the spleen. This procedure is indicated when a lesion is located in the tail of the pancreas. Some patients with chronic pancreatitis may also have indications for a distal pancreatectomy. However, most of these procedures are performed for tumor removal. Distal pancreatectomy has an overall mortality rate of 3% and morbidity of 47%. Most common serious adverse effect is pancreatic leak/fistula, and it occurs in around 30% of patients that undergo distal pancreatectomy.^17^

**Open pancreaticoduodenectomy**:

Also called Whipple procedure, entails the removal of the head of the pancreas, duodenum, a part of the stomach and the gallbladder. Surgical techniques have improved mortality rates of this procedure and large-scale studies estimate a mortality of 4%.^18^

**Indocyanine green**: is an FDA-approved dye which emits light when near-infrared light is applied to it. ICG can be injected intravenously or directly to certain tissues. When applied intravenously, ICG travels to tissues by arteries. During surgery, near-infrared light can be used to visualize an overlay of ICG over the normal image of the tissue. ICG then delimits the tissue exemplifying blood perfusion in that area. ICG use was first described more than 50 years ago. Notably, with a 1958 study describing its uptake and excretion in liver and biliary tracts.^19^ Since then, studies of ICG both in the lab and in clinical trials, have shown promising results of its use in different surgical procedures. Yearly published studies on PubMed for ICG have more than doubled from 2012 to 2022, with 651 and 1519 studies respectively. ICG has demonstrated an excellent safety profile in clinical trials among different gastrointestinal surgeries, including recently in pancreatic surgery.^20^

**ICG in surgery of the pancreas**:

ICG has been extensively used in surgical fields, including but not limited to colorectal, hepatic and gynecological surgery. However, pancreas surgery studies on ICG have been more limited. Recent studies on ICG use in pancreatectomies have been promising for this evolving field. There is one case-report of a single patient where hypoperfusion was seen with ICG after pancreatectomy and then developed a fistula.^21^ Consensus surveys show pancreas surgeons have a favorable outlook to ICG in procedures because it does not prolong surgical time and its great safety profile on patients. A cohort of pancreas surgeons recently expressed their interest in further studies regarding better visualization of tissue blood perfusion using ICG.^22^

**ICG for evaluation of leaks after pancreatectomy**:

To date, there has been only one study directly regarding the use of ICG for pancreatic leak after removal of the pancreas. However, their hypothesis was done retrospectively in only one patient after the surgery.^21^

## Study Rationale

Current methods of evaluation for poor tissue perfusion are not ideal. Surgeons rely on visualization of the pancreatic remnant and assessment of the color and texture of the pancreas. Recently use of ultrasound has aided as another measure for tissue perfusion. However, ultrasound is operator-dependent by nature and does not provide an objective parameter. Studies show poor correlation of visual data surgeon gathers and actual tissue perfusion.

Our hypothesis is that remnant pancreas perfusion will be easily and objectively evaluated with intravenous ICG followed by imaging.

- - 1. Known Potential Risks

An immediate risk that would be presented to patients with ICG administration is exposure to an agent that could cause an adverse reaction even if there is no previous personal history of it. Overall adverse reaction to systemic ICG is estimated to be around 5% with only 0.05% as serious adverse reactions.^23, 24^ The most common adverse reactions include nausea, extravasation of ICG and urticaria. As per FDA drug insert, anaphylaxis has been reported with use of ICG. Less than 10 confirmed cases of anaphylaxis by ICG under general anesthesia are available in current literature.^25^ Addition of ICG to a surgical procedure seems to add neglible risk for adverse effects to the baseline risk of the surgery itself.

- - 1. Known Potential Benefits

Potential benefits include the possibility of detecting low perfusion tissue during surgery

Long range potential benefits: if ICG uptake correlates with pancreatic remnant perfusion, critical information for avoiding hypoperfusion will be available for clinical decisions. This study aims to investigate the relationship between hypoperfusion in surgery and postoperative leak development. Currently, no measurable, modifiable intraoperative intervention is known to decrease leak rates. Our long-range goal is carrying forward a positive outcome from this study into a clinical trial evaluating improving remnant gland perfusion and whether this may decrease postoperative fistulae.

# OBJECTIVES AND ENDPOINTS

## Primary Objective and Endpoint

| **Primary Objective** | **Endpoint** | **Start** | **End** |
| --- | --- | --- | --- |
| To determine if altered measurement results of ICG after pancreatectomy is associated with leak rates. | Incidence of biochemical leak or fistula after pancreatectomy defined by definitions of the ISGPS criteria | Day of surgery | Measurement of drain fluid amylase in postoperative period up until day 30 |
|  | Perfusion status measured by ICG metrics | Day of surgery | Day of surgery |

## Secondary Objectives and Endpoints

| **Secondary Objective** | **Endpoint** | **Start** | **End** |
| --- | --- | --- | --- |
| To evaluate the grade of leak via ISGPS classification and ICG metrics | Leak grade via ISGPS classification | When a leak is detected | Last day of postoperative follow-up (day 30 after surgery) |
|  | ICG metrics | Day of surgery | Day of surgery |
| Determine best practices of usage of ICG for pancreatic surgery | Results of short verbal survey to surgeon and other OR members | During the first 15 measurements. | At surgery number 15 |

# STUDY DESIGN

## Description of the Study Design

*Refer to* Section 11*, Statistical Analysis for additional information regarding statistical methods used in this study.*

This is a Phase II study to assess diagnostic parameters of hypoperfused pancreatic cut edge via ICG and its relation to fistula formation versus conventional measurements. The study design consists of a single arm intervention group (n=50).

The trial will commence with the enrollment of the fifteen patients, after these 15 casesa logistics run-in of the OR ICG technique and timing will be done. During the logistics run-in, the PI will evaluate team satisfaction and measurement outcomes. This will be done through the application of a 3-question paper-based quality survey, attached in the supporting documents for the protocol. If an average score below 50% is given, study will be terminated to avoid negative impact on the logistics of pancreatectomies. Survey scores are calculated depending on the answer given, A= 0% B = 16.5% C = 33%. Minimum score 0 maximum score 100. Progressive study enrollment will continue throughout the adjustments, and enrollment will be halted if any major concerns arise. If the interim analysis does not result in study termination, enrollment of the remaining participants will resume to enroll a total of 50 patients completing study intervention and follow-up.

Aside from interim analysis evaluating logistics. A patient safety analysis will be performed at the same time. Herein, potential factors of the clinical trial that could negatively impact patient outcomes will be addressed. These factors include adverse effects of ICG administration and factors from the clinical trial process that negatively impact the conventional treatment course of patients. If any concerns are identified on interim safety analysis, the study could temporarily pause enrollment until the issues are addressed. This is to supplement study stopping rules delineated in 9.6 and Data and Safety forms.

The study schedule will be divided into 2 periods: (1) the intra-surgical period which starts at the time of ICG injection and ends when the surgical procedure is terminated and (2) the postoperative period which begins at the end of surgery and ends 30 days postoperatively.

We will use a cutoff of 60% of the maximum ICG uptake to consider cut edge as poorly perfused. (100% measured 4-6 cm from the cut edge of the pancreas point 5 and 6 in the figure below). Additionally, to compare with non-pathologic gastrointestinal tissue perfusion, ICG uptake metrics will be captured from the gastric body.


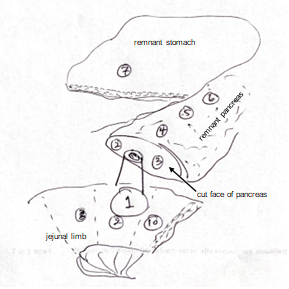


At the completion of the proposed study, the expected outcomes are to have evaluated the safety and efficacy of intra-operative ICG administration in the assessment of pancreatic perfusion at the cut edge.

## End of Study Definition

A participant is considered to have completed the study if he or she has completed all phases of the study including the last visit or the last scheduled procedure shown in the Schedule of Events (Section 7.11).

## Study Discontinuation and Closure

This study may be temporarily suspended or prematurely terminated if there is sufficient reasonable cause. Written notification that documents the reason for study suspension or termination will be provided by the suspending or terminating party to OHSU Coordinating Center, local IRB, and other regulatory authority (if required). If the study is prematurely terminated or suspended, the Investigator must promptly inform the IRB and provide the reason(s) for the termination or suspension. The OHSU Coordinating Center will notify sub-site(s) of any study suspension or discontinuation.

Reasons for terminating the study may include the following:

• Unfavorable assessment of risk/benefit ratio

• Incidence or severity of adverse events, in this or other studies, that indicates a

potential health hazard to participants

• Demonstration of lack of efficacy that warrants stopping

• Data that are not sufficiently complete and/or evaluable

• Investigator not adhering to the study protocol or applicable regulatory guidelines in conducting the study

• Participant enrollment is unsatisfactory

• Submission of knowingly false information from the study site to OHSU Coordinating Center or regulatory authority

• Upon instruction by local or other regulatory or oversight authority.

The study may resume once concerns about safety, protocol compliance, and/or data quality are addressed as applicable and requirements of the OHSU Coordinating Center, funder, IRB and/or other applicable regulatory authority are satisfied.

# STUDY POPULATION

## Participant Inclusion Criteria

To be eligible to participate in this study, an individual must meet all of the following criteria:

1. Participant scheduled for open pancreaticoduodenectomy or distal pancreatectomy for any diagnosis
2. Participant ≥ 18 years of age
3. Ability to understand nature and individual consequences of clinical trial
4. Written informed consent from participant or legally authorized representative.
5. For participants of childbearing potential, a negative pregnancy test and adequate contraception until 14 days after trial intervention
6. Participant needs to have an operative drain (any closed suction drain) after the procedure.
7. Participants that do not require arterial reconstruction
8. Participants that require minor portal venous recounstructions including patch venoplasty

## Participant Exclusion Criteria

An individual who meets any of the following criteria will be excluded from participation in this study:

1. Patients with previous history of adverse reaction to contraste dye, ICG or components of the dye
2. Prior pancreatectomy
3. Known diagnosis of hepatic insufficiency, hepatitis, liver fibrosis or cirrhosis, or chronic pancreatitis
4. Because this study focuses on hypoperfusion, patients will be excluded if in postoperative day 3-5 had any of the following: persistent SBP <90 mmHg unresponsive to 1L crystalloid, unexpected ICU transfer, blood transfusion of >2 units intraoperatively or 1 postoperatively, vasopressor treatment or ACLS protocol initiation
5. Organ failure, anuria or NSQIP-identified complication will be reviewed by PI and attending surgeon and excluded
6. Patients that require arterial reconstruction as part of their procedures

## Lifestyle Considerations

During this study, participants are not asked to modify lifestyle or diet apart from standard of care peri-procedural measures prior to distal pancreatic resection or open pancreaticoduodenectomy.

## Strategies for Recruitment and Retention

This study will be conducted in the United States. Participants for this study will primarily be recruited from GI and surgical practices within OHSU and its affiliated community partners, but additional collaborative study sites may also be invited to participate in this trial. Participants may be identified and referred to this study by their primary treating physician from within OHSU, collaborating study sites, or from the outside community. Participants may be identified by a member of the participant’s treatment team, the PI, research team, or medical and surgical oncology clinics part of OHSU or collaborating study sites. As a member of the treatment team, the investigator(s) will screen their participant’s medical records for suitable research study participants and discuss the study and their potential for enrolling in the research study. Referral of potential participants to investigator(s) of this study is made as part of standard of care, with the referring physician seeking advice on the diagnosis, evaluation, and/or treatment of the participant’s malignancy.

The investigator(s) may also screen the medical records of potential participants with whom the investigator does not have a treatment relationship. This will be done for the limited purpose of identifying patients who would be eligible to enroll in the study and to record appropriate contact information in order to approach these potential individuals regarding the possibility of participating in the study. Participants may also initiate contact with the investigator through information of this study posted on the clinicaltrials.gov website.

- - 1. Accrual Estimates

Total accrual of all participants is anticipated to take a total of 12 months.

No OHSU Knight Cancer Institute study will focus on any particular gender, racial or ethnic subset. No participant will be excluded from the study on the basis of gender, racial or ethnic origin. Male, female and minority volunteers will be recruited for this study from the general population and approximately 50% men and 50% women will be studied. Gender-nonconforming and gender-fluid individuals as members of the general population will also be recruited. Pregnant persons, decisionally impaired adults and prisoners are excluded from this study.

| **Table 1.** **Population Demographics - Oregon (%)** | | | | | |
| --- | --- | --- | --- | --- | --- |
| **Ethnic Category** | **Sex/Gender** | | | | |
|  | Females | | Males | | Total |
| Hispanic or Latino |  | 6.6 |  | 6.5 | 13.1 |
| Not Hispanic or Latino |  | 43.8 |  | 43.1 | 86.9 |
| **Ethnic Category: Total of all participants*** |  |  |  |  | 100* |
|  | | | | | |
| **Racial Category** |  | | | | |
| American Indian or Alaskan Native |  | 0.9 |  | 0.9 | 1.8 |
| Asian |  | 2.4 |  | 2.3 | 4.7 |
| Black or African American |  | 1.1 |  | 1.1 | 2.2 |
| Native Hawaiian or other Pacific Islander |  | 0.2 |  | 0.2 | 0.4 |
| White |  | 43.9 |  | 43.2 | 87.1 |
| Two or more races |  | 1.9 |  | 1.9 | 3.8 |
|  | | | | | |
| **Racial Category: Total of all participants*** |  | |  | | 100* |
| TOTALS |  | 50.4 |  | 49.6 | 100* |
| **Source:** Adapted from U.S. Census Bureau, 2017.  *Totals may not equal 100 due to rounding. | | | | | |

**Table 2. Projected Accrual for the Present Study**

| **Ethnic Category** | **Sex/Gender** | | | |
| --- | --- | --- | --- | --- |
|  | Females | Males | Other/Unknown | Total |
| Hispanic or Latino | 3 | 3 |  | 6 |
| Not Hispanic or Latino | 22 | 21 |  | 22 |
| Unknown | 0 | 0 |  | 0 |
| **Ethnic Category: Total of all participants*** | 25 | 24 |  | 49* |
| **Racial Category** |  | | | |
| American Indian or Alaskan Native | 0-1 | 0-1 |  | 0-2 |
| Asian | 1 | 1 |  | 2 |
| Black or African American | 1 | 1 |  | 2 |
| Native Hawaiian or other Pacific Islander | 0-1 | 0-1 |  | 0-2 |
| White | 22 | 21 |  | 43 |
| More than one race | 1 | 1 |  | 2 |
| Unknown | 0-1 | 0-1 |  | 0-2 |
| **Racial Category: Total of all participants*** | 28 | 27 |  | 55* |
| **Source:** Adapted from U.S. Census Bureau, 2017.  *Totals may not equal 100 due to rounding. | | | | |

- - 1. Inclusion of Children

This protocol does not include children for the following reason:

1. The number of children with this type of cancer/condition is limited

# PARTICIPANT SCREENING, ENROLLMENT, AND WITHDRAWAL

This is a single-arm phase II clinical trial (without randomization).

Each individual that consents to participate in this study must be entered into OHSU’s electronic clinical research information system, eCRIS, regardless of Screening outcome.

## Consent and Screening

In order to participate in this study, signed informed consent must be obtained from the participant or the participant's legally acceptable representative. The current IRB approved informed consent form must be signed and dated by each participant prior to undergoing any study procedures or before any prohibited medications are withheld from the participant in order to participate in this study. The informed consent discussion must be documented, and a copy of their signed IRB approved informed consent form must be scanned in the participant’s medical record.

Each site will maintain a screening log of all participants who are approached for the study, as well as source documentation related to the consent and screening outcome for each, including an explanation for exclusion due to screen failure where applicable. Each site is required to retain, in a confidential manner, sufficient information on each participant so that the participant may be contacted should the need arise.

For consenting subjects with Limited English Proficiency, a short consent will be provided in the subject’s native language. Consenting for patients with limited English proficiency will only be done by a member of the study team if said member is certified by OHSU’s language services to interact with patients in the corresponding language.

For languages other than English and if the study team member is not certified to interact with patients in that language directly, a short form of consent in the subject’s native language will be used only when an interpreter is present. Short forms must be in the subject’s native language. If no interpreter is available, consent for Limited English Proficiency participants will not happen.

- - 1. Screening Period

The screening period begins once the participant has provided written informed consent to participate in the study and ends once ICG injection is initiated. All screening and baseline evaluations will be performed during the screening period. Day 1 of the clinical trial will be when participants are started on the study intervention.

- - 1. Re-testing During Screening

Re-testing of laboratory parameters and/or other assessments within the Screening or Lead-in period will be permitted (in addition to any parameters that require a confirmatory value).

The most current result prior to Enrollment is the value by which study inclusion will be assessed, as it represents the participant’s most current, clinical state.

Laboratory parameters and/or assessments may be repeated in an effort to find all possible well-qualified participants. Consultation with the Principal Investigator may be needed to identify whether repeat testing of any particular parameter is clinically relevant.

## Enrollment Procedures

This is a Phase II trial, and there is no randomization for treatment.

- - 1. Enrollment Procedures for the OHSU Site

Eligibility must be confirmed and documented by the Principal Investigator prior to enrollment.

Materials required to complete the eligibility review include, at minimum:

• Current IRB-approved consent form and HIPAA Authorization for the study signed & dated by the participant

• Documented (signed and dated) attestation by the PI confirming participant’s eligibility based on available source documentation and authorizing enrollment

• Direct surgical evaluation at the time of planned study intervention to verify anatomic eligibility for ICG injection

Once eligibility is confirmed, the participant is considered ‘enrolled’ once study intervention is performed.

## Measures to Minimize Bias

- - 1. Randomization/Masking Procedures

While no randomization of patients will be performed, surgeons will be blinded to ICG perfusion measurements. To avoid undue revision or operative conduct should the gland demonstrate any characteristics (perceived or real) of hypoperfusion, surgeon blinding is key. The blinding will be performed by capturing and analyzing the perfusion images out of view of all non-study personnel obtaining images. Doppler blinding will be conducted using a tape recorder attached to the Doppler unit which disables the speaker.

- - 1. Evaluation of Success of Blinding

A dedicated staff member will evaluate for adequate blinding of the surgeon(s) during perfusion measurements. Measurement of target perfusion tissue with Spy Elite device will only be done when the operator is certain that the surgeon is not watching the device’s screen. The success or failure of adequate blinding will be recorded for each procedure.

The Spy Elite device has a small articulated monitor that will be set up to be facing away from the surgeon. ICG measurements can only be seen on the device monitor and not on other screens in the OR.

If concerns for blinding are noted by the study personnel, these instances will be noted. If no adequate blinding could be achieved ie. There is suspicion that the surgeon saw any of the measurements, these cases will not be used for analysis.

All the surgeons participating in the study are aware of the importance of blinding and that violation of blinding impairs the analysis.

- - 1. Breaking the Study Blind/Participant Code

Because intervention of ICG does not currently change clinical practice, there is no need to break the blinding protocol to the surgeon performing the procedure. Blinding of patients does not apply.

## Screen Failures

Screen failures are defined as participants who consent to participate in the clinical trial but are not subsequently assigned to the study intervention or entered in the study. A minimal set of screen failure information is required to ensure transparent reporting of screen failure participants, to meet the Consolidated Standards of Reporting Trials (CONSORT) publishing requirements and to respond to queries from regulatory authorities. Minimal information includes demography, screen failure details, eligibility criteria, and any serious adverse event (SAE).

Individuals who do not meet the criteria for participation in this trial (screen failure) because of a failing to meet eligibility criteria may be rescreened. Rescreened participants should be assigned the same participant number as for the initial screening.

Any participant that has signed the consent form (for either screening or study participation) but does not meet all of the study eligibility criteria, or meets study eligibility criteria but terminates their participation prior to receiving study treatment, will be considered a screen failure and not counted towards total number of planned enrollments. The reason for screen failure should be captured in the research record for each participant who fails to meet all of the eligibility criteria.

- - 1. Re-Screening Allowance / Procedures

This study permits the re-screening of a participant that has discontinued the study as a screen failure (i.e., participant has not been treated). If re-screened, the participant must be re-consented.

## Participant Discontinuation or Withdrawal

Participants are free to withdraw consent and discontinue participation in the study at any time and without prejudice to further treatment. If a participant no longer wants to receive investigational product, but is willing to come for follow-up appointments, the participant’s request should be honored, if possible.

If a participant withdraws consent, they should be asked to specify if they are withdrawing consent to all further participation in the study, including any further follow up (e.g., survival contact telephone calls) or if they are choosing to withdraw only from further study intervention, meaning that further follow-up and data collection about their disease and health status is allowable. The participant should also be asked about their consent to the future use of their study-generated data and any biological samples, as applicable.

No further participant contact should be made if the participant withdraws consent for participation in the study. Information about the reason(s) for discontinuation and collection of any new or ongoing AEs should be collected at the time the participant withdraws consent.

A participant may also be withdrawn from the study by the Sponsor/Sponsor-Investigator, Investigator, local IRB, or regulatory authorities.

Reasons for a participant to discontinue the study may include the following:

• Participant dies or is lost to follow-up

• Participant withdraws consent for any further participation

• The end of study is reached

• Pregnancy

• Significant study intervention non-compliance

• If any clinical adverse event (AE), laboratory abnormality, or other medical condition or situation occurs such that continued participation in the study would not be in the best interest of the participant

• Disease progression which requires discontinuation of the study intervention

• If the participant meets an exclusion criterion (either newly developed or not previously recognized) that precludes further study participation

In the event of a pregnancy, the study treatment will not be administered. Refer to Section 10.6.5 regarding reporting of pregnancy. Participants who withdraw or discontinue after receiving study intervention count towards the enrollment total.

- - 1. Handling Participant Discontinuation from Study

When a participant discontinues participation in the study, the reason the participant is no longer participating, the study name, IRB study number, and the date of discontinuation must be documented in the participant’s medical record. The change in study status must be documented in the appropriate clinical trial management system (e.g., eCRIS) per OHSU policy.

For all other reasons for discontinuation from the study treatment phase, the participant should return to the clinic for the end of treatment (EOT) visit according to Section 8, STUDY PROCEDURES/EVALUATIONS AND SCHEDULE

## Lost to Follow-up

A participant will be considered “lost to follow-up” if the participant:

Undergoes ICG injection and planned surgical intervention but does not present for planned post-operative follow-up appointment and is unable to be contacted by study staff for 30 days following postoperative hospital discharge.

The following actions must be taken if a participant fails to return to the clinic for a required study visit:

- The site will attempt to contact the participant and reschedule the missed visit within two weeks, and counsel the participant on the importance of maintaining the assigned visit schedule and ascertain if the participant wishes to and/or should continue in the study.
- Before a participant is deemed lost to follow-up, the investigator or designee will make every effort to regain contact with the participant (where possible, 3 telephone calls and, if necessary, a certified letter to the participant’s last known mailing address or local equivalent methods). These contact attempts should be documented in the participant’s medical record or study file.
- Should the participant continue to be unreachable, he or she will be considered to have withdrawn from the study with a primary reason of lost to follow-up.

# STUDY INTERVENTION

A list of the adverse events and potential risks associated with the study intervention administered in this study can be found in Section 10.4, Adverse Events.

## Name of Study Intervention

- - 1. Study Intervention Description

**Indocyanine green**: ICG is an FDA-approved intravenous drug that binds albumin and when detected using near-infrared imaging performs well as a marker of blood flow and oxygenation.^26^ When applied intravenously, ICG travels to tissues by arteries. ICG has been used for more than 50 years. ^19^ Since then, studies of ICG both in the lab and in clinical trials, have shown promising results of its use in different surgical procedures. ICG has an excellent safety profile and adds little to no risk to patients undergoing surgery.^23, 24^

Study Device information is detailed in section 6.2, but briefly includes use of the Stryker Spy+ Elite platform. The Stryker Spy+ Elite is an FDA approved imaging system for intraoperative acquisition of near-infrared images and measurement of ICG perfusion kinetics and metrics. ICG uptake data will be measured immediately before reconstruction in the case of pancreaticoduodenectomies and for distal pancreatectomies, during visual inspection of right upper quadrant prior to surgical closure.

- - 1. Acquisition

ICG for Injection is on OHSU Pharmacy Formulary and comes as a single-patient vial of 25mg of lyophilized drug, which is then reconstituted with 10mL of sterile injectable saline.

- - 1. Formulation, Appearance, Packaging and Labeling

ICG is an FDA approved drug supplied as a sterile, lyophilized green powder containing 25 mg of indocyanine green with no more than 5 % sodium iodide. It is packaged with an Aqueous Solvent consisting of Sterile Water for Injection used to dissolve the indocyanine green. ICG is to be administered intravenously.

- - 1. Product Storage and Stability

ICG should be stored at 15° to 25° C.

ICG should be used within 6 hours after it is prepared.

- - 1. Compatibility

ICG should only be prepared with the included aqueous solvent.

- - 1. Handling

FDA data states that ICG is unstable in aqueous solution and must be used within 6 hours. However, the dye is stable in plasma and whole blood so that samples obtained in discontinuous sampling techniques may be read hours later. Sterile techniques should be used in handling the dye solution as well as in the performance of the dilution curves.

- - 1. Preparation

Under sterile conditions, reconstitute one (1) 25 mg vial of Indocyanine Green for Injection, USP using one (1) 10 mL Sterile Water for Injection, USP vial located in the ICG for Injection Set. Shake the ICG vial gently to dissolve. After reconstitution, a 25 mg vial of ICG contains 2.5 mg of dye per mL of solution, so a 1.0 mL injection contains a 2.5 mg dose of ICG.

Indocyanine Green for Injection, USP must be used within 6 hours after reconstitution. If a precipitate is present, discard the solution.

- - 1. Administration

ICG is for intravenous use. All administration of ICG as part of this study will be through a peripheral intravenous line or a central line placed at the discretion of the anesthesiologist for the purpose of medication administration as a standard part of the operation. Administration of the dye plus measurement of tissue perfusion is an additional step that is not done in conventional pancreatectomies. Based on studies looking at ICG use in other gastrointestinal surgeries, we can expect an increase of operative time of 10-15 minutes. Data shows that this increase in operative time when using ICG versus not using this technology is not statistically significant. ^26,27^ Study staff in the operating room will measure the time to get information on the impact of normal workflow of these surgeries. Additionally, need of repeat doses of ICG will be recorded and compared to operative time to measure the impact. Upon completion of the study, descriptive statistics will be reported to analyze the impact of ICG use and perfusion measurements on pancreatic surgeries.

- - 1. Special Considerations for Administration

Heparin preparations containing sodium bisulfate reduce the absorption peak of ICG in blood and, therefore, should not be used as an anticoagulant for the collection of samples for analysis. Drug/Laboratory Test Interactions: Radioactive iodine uptake studies should not be performed for at least a week following the use of ICG.

During IV administation of ICG, vital signs will be continously monitored for any change from normal parameters. All subjects will have their heart rate, respiratory rate, oxygen saturations, blood pressure and ventilation will be monitored throughout and after the procedure. If subjects develop hypersensitivity symptoms like rash, wheezing, or pruritus, hypersensitivity medications should be administered.

There are no other special considerations, including hydration, steroids, special IV tubing, filtration, or special equipment required.

Physician administering IV ICG must wear sterile gloves and use sterile technique when using ICG.

- - 1. Accountability

The Investigator, or a responsible party designated by the Investigator, must maintain a careful record of the inventory and disposition of the study agent. (See the [NCI Investigator’s Handbook for Procedures for Drug Accountability and Storage](http://ctep.cancer.gov/investigatorResources/investigators_handbook.htm)).

As this protocol specifies an investigational application of an FDA approved substance which is on-formulary at OHSU, storage, tracking, inventory, and return will be tracked by the OHSU Pharmacy as per current standard operating practices. Utilized drug details including but not limited to dose, expiration date, manufacturing date, and lot number will be recorded for participants.

Responsibility for drug accountability at the study site rests with the Investigator; however, the Investigator may assign some of the drug accountability duties to an appropriate pharmacist or designee. Inventory and accountability records must be maintained and readily available for inspection by the study monitor and are open to inspection at any time by any applicable regulatory authorities or other oversight bodies.

The Investigator or designee will collect and retain all used, unused, and partially used containers of study medication until full accounting has been completed. The Investigator or designee must maintain records that document:

- Investigational product delivery to the study site.
- The inventory at the site.
- Use by each participant including pill/unit counts from each supply dispensed.
- Return of investigational product to the Investigator or designee.
- Destruction or return of investigational product for final disposal.

These records should include dates, quantities, batch/serial numbers (if available), and the unique code numbers (if available) assigned to the investigational product and study participants.

The investigational product must be used only in accordance with the protocol. The Investigator will also maintain records adequately documenting that the participants were provided the correct study medication specified.

Completed accountability records will be archived by the site.

## Device Specific Considerations

Novadaq Stryker Spy Elite will be used, specifically model number LC3000. This device is regulated by FDA code 21 CFR § 892.1600. Exposure to the imaging device is limited to less than five minutes. Measurement usually takes a couple of minutes, and it is used after ICG IV administration. Measurements will be done only once to patients.

Software for measurement includes CINEVAQ 2.0 or newer, contained in the SPY Elite device. This allows for an interface for image acquisition, processing and controls of the device.

# TREATMENT PLAN

## Dosage and Administration

| **Regimen Description** | | | | | |
| --- | --- | --- | --- | --- | --- |
| *Agent* | *Premedication; Precautions* | *Dose* | *Route** | *Schedule* | *Cycle Length* |
| Indocyanine green | none | 5.0 mg | IV | During surgery | Single injection; may be repeated to maximum dose of 2mg/kg |
| **Specify route of administration (e.g., IVB, IVP, IVCI, PO, intra-arterial, peritoneal, intrathecal, intracavity, etc.) and duration (e.g., IVP over 10 seconds), including any allowable time windows (+/- minutes).* | | | | | |

Repeated doses of ICG could be considered if the initial dose does not create adequate uptake in reference tissue when creating a baseline to then compare with the cut edge of the pancreas. Adequate uptake will be defined as a change of ICG detected by the Spy Elite+ device when compared to tissue before ICG administration. Repeated doses of ICG injections while staying under the maximum dose would not significantly increase risk for adverse reactions. However, requiring additional doses of the dye could increase total operative time. Impact of operative time with use of ICG is further addressed in 6.1.8. “ADMINISTRATION”.

## Concomitant Medication and Supportive Care Guidelines

Supportive measures for optimal medical care are to be given throughout the study as indicated by the treating physician’s assessment of the participant’s medical need and institutional and general medical guidelines for the care of participants undergoing pancreaticoduodenectomies or distal pancreas resections.

Medications required to treat AEs, manage cancer symptoms, concurrent diseases and supportive care agents, such as pain medications, anti-emetics and anti-diarrheals are allowed in general. The participant must be told to notify the investigational site about any new medications begun after the start of the study treatment.

All medications (other than investigational products) and significant non-drug therapies (including vitamins, herbal medications, physical therapy and blood transfusions) administered during the study must be listed on the case report form (CRF).

Treatment recommendations described in the following sections may be modified according to institutional standards and guidelines as appropriate.

## Precautionary Medications, Treatments, and Procedures

To the extent possible haloperidol, heparin, nifedipine, nitrofurantoin, phenobarbital and primidone are to be avoided because of their effects on ICG pharmacokinetics.

## Prohibited Medications, Treatments, and Procedures

Active vasopressor requirement during administration will be considered in final analysis and potential exclusion, however no specific medications, treatments, or procedures are considered “prohibited” for the purposes of this protocol.

# STUDY PROCEDURES/EVALUATIONS AND SCHEDULE

## Study-Specific Procedures

- - 1. Medical history

A medical history will be obtained by the investigator or qualified designee. In addition to collecting information on demographics, the medical history will include all active conditions, and any condition diagnosed within the prior 10 years that are considered to be clinically significant by the Investigator. Details regarding the participant’s malignancy, when applicable, will be recorded separately and not listed as medical history (e.g., date of diagnosis, staging).

- - 1. Disease Assessment

The investigator or qualified designee will obtain prior and current details regarding the participant’s pancreatic pathology.

- - 1. Medication review

A complete medication history will be acquired concurrent with medical history.

- - 1. Physical examination

Physical exams must be performed by a medically qualified individual such as a licensed physician, Physician’s Assistant, or Advanced Registered Nurse Practitioner as local law permits and per institutional standards. The physical examination to be conducted will include an evaluation of: general appearance; head, ears, eyes, nose, and throat; neck; skin; cardiovascular system; respiratory system; gastrointestinal system; lymphatic system, musculoskeletal system, and nervous system. All other physical exams after baseline will include an evaluation of any AEs, or any previously reported symptoms, or prior physical examination findings. All physical examinations will also include:

Vitals to be collected include blood pressure (BP), heart rate (HR), temperature, and oxygen saturation by pulse oximetry. As part of screening/baseline visit, vitals should be obtained prior to the surgery. Vitals will also be obtained during surgery and administration of ICG.

Significant findings that were present prior to the signature of the informed consent must be included in the Medical History eCRF page. Significant new findings that begin or worsen after informed consent must be recorded on the Adverse Event eCRF page.

Height will be collected at screening only. Weight will be collected at each in person visit.

Performance status (Eastern Cooperative Oncology Group) will be determined for all participants at screening.

- - 1. Radiographic or other imaging assessments

Conventional imaging for staging of pancreatic malignancy will be performed to all screened patients. During ICG administration, the NOVODAQ Spy+ Elite Platform will be used to capture reference and pancreatic perfusion, utilizing platform software.

- - 1. Adverse event evaluation

Toxicities and adverse experiences will be assessed using the [NCI CTCAE 5.0](http://ctep.cancer.gov/protocolDevelopment/electronic_applications/ctc.htm). Safety will be monitored by assessing physical examination, vital signs, body height (screening only) and weight as clinically indicated. Additionally, hematology, chemistry, coagulation, urinalysis, thyroid function, and pregnancy will be assessed as clinically indicated.

Adverse events will be monitored from the time the participant receives treatment on study. Participants will be instructed to report all AEs during the study and will be assessed for the occurrence of AEs throughout the study, including and up to 30 days. Due to the known excellent safety profile of ICG, AEs grade 1-2 will not be collected during the study. AEs Grade 3 or higher must be recorded on the source documents and eCRFs regardless of the assumption of a causal relationship with the study drug.

Abnormal laboratory values will only be recorded as an AE if determined to be clinically significant by the investigator.

- - 1. Counseling procedures

As the impact of ICG perfusion is yet unknown with respect to clinical outcomes, results will be provided to the participants as an aggregate of overall outcomes when the study is analyzed. Individual patient measurements will be used for research purposes only and patients will be apprised of this condition during the consent process.

- - 1. Assessment of participant-reported outcomes

A survey will be answered by registered nurses, technicians and surgeons after the surgery to better understand subjective assessments of ICG use concomitant with Spy Elite platform and software.

## Laboratory Procedures and Evaluations

HEMATOLOGY

Only as clinically indicated per institutional standard of care.

COMPREHENSIVE METABOLIC PANEL

Only as clinically indicated per institutional standard of care.

COAGULATION PANEL

Only as clinically indicated per institutional standard of care.

PREGNANCY TEST

A serum or urine pregnancy test is required during screening for all persons of childbearing potential. The pregnancy test is required within 7 days prior to study intervention and results must be available prior to administration of study agent. If the urine pregnancy test is positive, a serum pregnancy test must be performed per institutional standards.

AMYLASE ASSAY

Amylase levels will be drawn from all surgical drains, when present, on postoperative day 3 or 4±4, per standard of care prior following pancreas resections. In patients undergoing percutaneous drainage of a postoperative intra-abdominal fluid collection, amylase levels on the drained fluid will be drawn to evaluate for fistula. An amylase level >3 times the upper limit of normal from a surgical drain is sufficient for diagnosis of leak. Serum amylase will also be collected on the day drain amylase is collected as a referent.

## Biomarker, Correlative, and Special Studies

N/A

## Screening Assessments

A screening (consultation) visit may occur as part of standard of care. If a participant is eligible for the study after review of key inclusion/exclusion criteria, additional screening visits will be scheduled while staff members are requesting insurance authorization to participate in a clinical trial.

The following will be reviewed at screening visit:

• Clinical history and physical exam (per standard of care)

• Informed consent obtained and documented

Toxicities which occur prior to the start of treatment will not be subject to analysis. Consent must be obtained before initiation of any clinical screening procedure that are performed solely for the purpose of determining eligibility for this research study. Evaluations performed as part of routine care before informed consent can be utilized as screening evaluations if done within the defined time period.

## Baseline Assessments

Baseline screening evaluations are to be conducted within 4 weeks prior to start of protocol treatment. Imaging must be done within 4 weeks prior to start of protocol treatment.

## Early Termination or End of Treatment Visit

Any participant who undergoes early termination will undergo end of study visits depending on their progress in the protocol. Participants who have not received ICG injection are not considered enrolled, will not require a follow up visit and will be treated per standard of care. Participants undergoing termination following pancreatectomy will have an end of study visit at the time of their regular post-op visit and will be cared for per the standard of care.

## Follow-up

Per standard of care, participants will be followed by their attending surgeon and team postoperatively. Study follow-up will include acquisition of NSQIP outcomes, collected for the 30 days following pancreas resection for efficacy and safety of ICG. A follow-up visit will occur 14 days (± 7 days)-following hospital discharge, consistent with standard of care practices. Additionally, all subjects will be contacted by phone by a NSQIP nurse reviewer for approximately 30 days following the operation to collect standard NSQIP data, which is outside of the scope of this protocol.

## Unscheduled Visits

Unscheduled study visits may occur at any time if medically warranted. Any assessments performed (e.g., laboratory or clinical assessments) at those visits that, in the opinion of the investigator, are deemed related to this study should be recorded in the CRF.

## Schedule of Events

| Table X. Schedule of Procedures and Evaluations | | | | | | |
| --- | --- | --- | --- | --- | --- | --- |
| **Visit Days (± 3 Days)** | **Screening** | **Surgery** | **Postop day 3** | **Discharge day** | **Postop day 30** | **Follow-Up** |
|  | **Days**  **-28 to -1** | **Day 1** | **Day 4±3** | **Day +-14** | **Day 14-30** | **0-30 Days post surgery** |
| Informed consent | X |  |  |  |  |  |
| Inclusion/exclusion criteria | X |  |  |  |  |  |
| Medical history (including weight loss history within the last 6 months) | X |  |  |  |  |  |
| Prior/concomitant medications | X | X |  | X |  |  |
| Height and weight | X | X |  | X | X |  |
| Comprehensive physical examination | X |  |  |  |  |  |
| Physical Examination | X | X |  | X | X | X |
| Vital signs | X | X | X | X | X |  |
| ECG (if indicated) | X | X |  |  |  |  |
| Pregnancy test (if indicated) | X | X |  |  |  |  |
| Routine blood draw (1 tablespoon) |  | X | X |  |  |  |
| Review any adverse events |  | X |  |  |  |  |
| Review all medications |  | X |  |  |  |  |
| ICG |  | X |  |  |  |  |
| Pancreas resection |  | X |  |  |  |  |
| Follow-up data collection |  |  |  |  |  | X |
| Amylase from drain fluid |  |  | X |  |  |  |
| ***FOOTNOTES:*** | | | | | | |

# EFFICACY MEASURES

Diagnostic efficacy will be evaluated in this study by the presence of any leaks and its relation with intraoperative tissue data.

## Definition of Efficacy Measures

Signs of postoperative fistula

Amylase level 3x the upper limit of normal from drain fluid.

## Disease Evaluation

Evaluation for fistula formation is done via amylase fluid analysis on postoperative drain and clinical suspicion of its classical symptoms.

## ISGPS DEFINITION AND GRADING OF POSTOPERATIVE PANCREATIC FISTULA

We will grade each patient’s postoperative characteristics via the ISGPS grading system. Bile Leak (previously grade A): increased drain amylase activity > 3 times the upper limit of institutional normal serum amylase activity. Grade B: bile leak criteria plus any of the following: persisting peripancreatic drainage >3 weeks, clinically relevant change in management of POPF, POPF percutaneous or endoscopic specific interventions for collections, angiographic procedures for POPF related bleeding. Grade C: whenever Grade B fistula requires reoperation, has signs of infection related to POPF with organ failure, POPF related organ failure or POPF related death.^28^

# SAFETY

## Specification Of Safety Parameters

The Investigator is responsible for monitoring the safety of participants who have enrolled in the study. Safety assessments will be based on medical review of adverse events and the results of safety evaluations at specified time points as described in Section 8.10, Schedule of Events. Any clinically significant adverse events persisting at the end of treatment visit will be followed by the Investigator until resolution/stabilization or death, whichever comes first.

## Definitions

- - 1. Adverse Event (AE)

An adverse event is defined as any undesirable physical, psychological or behavioral effect experienced by a participant during their participation in an investigational study, in conjunction with the use of the investigational product, whether considered intervention-related (21 CFR 312.32 (a)). In general, this includes signs or symptoms experienced by the participant from the time of signing the informed consent to completion of the study.

AEs may include, but are not limited to:

- Subjective or objective symptoms spontaneously offered by the participant and/or observed by the Investigator or medical staff.
- Clinically significant laboratory abnormalities.
- A significant worsening of the participant’s condition from study entry.
- Disease signs and symptoms and/or laboratory abnormalities existing prior to the use of the study treatment that resolve but then recur after treatment.
- Disease signs and symptoms and/or laboratory abnormalities existing prior to the use of the study treatment which increase in frequency, intensity, or a change in quality after treatment.
  - 1. Serious Adverse Event (SAE)

An AE or suspected adverse reaction is considered "serious" if, in the view of either the Investigator or Sponsor-Investigator, it results in any of the following outcomes:

- Death,
- A life-threatening adverse event,
- In-patient hospitalization or prolongation of existing hospitalization,
- A persistent or significant incapacity or substantial disruption of the ability to conduct normal life functions, or
- A congenital anomaly/birth defect.
- GI bleeding or perforation requiring hospitalization
- Any procedure-related complications resulting in delay of adequate and timely discharge of hospital
  - 1. Unanticipated Problems (UP)

The Office for Human Research Protections (OHRP) considers UPs involving risks to participants or others to include, in general, any incident, experience, or outcome that meets all of the following criteria:

1. Unexpected in terms of nature, severity, or frequency given (a) the research procedures that are described in the protocol-related documents, such as the IRB-approved research protocol and informed consent document; and (b) the characteristics of the participant population being studied;
2. Related or possibly related to participation in the research (“possibly related” means there is a reasonable possibility that the incident, experience, or outcome may have been caused by the procedures involved in the research); and
3. Suggests that the research places participants or others at a greater risk of harm (including physical, psychological, economic, or social harm) than previously known or recognized.

This study will use the OHRP definition of UP.

- - 1. Severity of Event

The Investigator will grade the severity of each AE using, when applicable, the current version of the [CTCAE v5.0](https://safetyprofiler-ctep.nci.nih.gov/CTC/CTC.aspx). In the event of an AE for which no grading scale exists, the Investigator will classify the AE as defined below:

| **Grade 1:** | Mild; asymptomatic or mild symptoms; clinical or diagnostic observations only; intervention not indicated. |
| --- | --- |
| **Grade 2:** | Moderate; minimal, local or noninvasive intervention indicated; limiting age-appropriate instrumental ADL |
| **Grade 3:** | Severe or medically significant but not immediately life-threatening; hospitalization or prolongation of hospitalization indicated; disabling; limiting self-care ADL. |
| **Grade 4:** | Life-threatening consequences; urgent intervention indicated. |
| **Grade 5:** | Death related to AE. |
| *Note: a semi-colon indicates ‘or’ within the description of the grade.* | |

- - 1. Assessment of Causality Relationship To Study intervention

For all collected AEs, the clinician who examines and evaluates the participant will determine the AE’s causality based on temporal relationship and his/her clinical judgment. The degree of certainty about causality will be graded using the categories below:

| **Definitely Related:** | There is clear evidence to suggest a causal relationship, and other possible contributing factors can be ruled out. |
| --- | --- |
| **Possibly Related:** | There is some evidence to suggest a causal relationship. |
| **Unrelated:** | The AE is completely independent of study intervention administration, and/or evidence exists that the event is definitely related to another etiology. |

## Expectedness

The Investigator will be responsible for determining whether an AE is expected or unexpected. An AE will be considered unexpected if the nature, severity, or frequency of the event is not consistent with the risk information previously described for the study agent.

## Adverse Event List(s)

- - 1. Adverse Event List for ICG INJECTION

Detailed information about the risks and expected AE of ICG may be found in the current edition of the manufacturer’s information brochure. In brief, the most frequent adverse reactions were allergic reactions with nausea or hypotension. For more detailed information refer to 1.4.1.

## Time Period And Frequency For Event Assessment And Follow-Up

The occurrence of an UP, AE or SAE may come to the attention of study personnel during study visits and interviews of a study participant presenting for medical care, upon review by a study monitor, or during an audit. All AEs grade 3 or higher but not meeting the criteria for SAEs will be captured on the appropriate CRF. AEs of grade 1 and 2 will not be captured due to the well-characterized side effect profile of ICG for other indications, including IV ICG injection which is FDA approved. Information to be collected includes event description, time of onset, clinician’s assessment of severity, seriousness, expectedness, relationship to study product (assessed only by those with the training and authority to make a diagnosis), and date of resolution/stabilization of the event. All AEs grade 3 or higher occurring while on study must be documented appropriately regardless of relationship. All AEs will be followed to adequate resolution.

Any medical condition that is present at the time that the participant is screened will be considered as baseline and not reported as an AE. However, if the study participant’s condition deteriorates at any time during the study, it will be recorded as an AE after treatment with study intervention begins.

Changes in the severity of an AE will be documented to allow an assessment of the duration of the event at each level of severity to be performed. AEs characterized as intermittent require documentation of onset and duration of each episode.

At each study visit, the Investigator will inquire about the occurrence of AE/SAEs since the last visit. Events will be followed for outcome information until resolution or stabilization.

The Investigator will record all reportable events with start dates occurring after treatment with study intervention begins for 30 days after date of surgery. AEs will be evaluated using the current version of the [CTCAE v5.0](https://safetyprofiler-ctep.nci.nih.gov/CTC/CTC.aspx). At each study visit, the investigator will inquire about the occurrence of AE/SAEs since the last visit. Events will be followed for outcome information until resolution or stabilization. Any SAE that occurs after treatment with alternative therapy will be reported only if the Investigator or current treating physician has assessed the SAE as related to the study treatment.

## Reporting Procedures

- - 1. OHSU IRB Reporting of Unanticipated Problems and Adverse Events

Unanticipated Problems and AEs will be reported to OHSU IRB according to the policies, procedures and guidelines posted on the [OHSU IRB web site](http://www.ohsu.edu/xd/about/services/integrity/policies/all-irb-documents.cfm).

Events that must be reported by the Investigator to the IRB are detailed in the OHSU IRB **Investigator Guidance: Prompt Reporting Requirements (HRP-801)**. Events that meet the criteria for OHSU RNI must be reported to the IRB within 5 days of learning of the event. At a minimum, events requiring reporting to the IRB include:

- Any new or increased risk related to the research, including AEs or IND safety reports that require a change to the protocol or consent,
- New FDA black box warning,
- Publications identifying new risks,
- Data Safety Monitoring Board/Committee letters recommending changes or discussing new risks
- Unanticipated adverse device effect
- Unauthorized disclosure of confidential participant information
  - 1. Central Reporting of Adverse Events for Multi-Site Studies

A sub-site must notify the OHSU Coordinating Center of any SAE by phone, fax, or email no later than 24 hours after learning of the event. The sub-site must also report each event to the institution’s local IRB or other oversight entity per institutional policies/requirements. The sub-site will send the Coordinating Center supporting materials regarding the SAE, as well as [describe what sub-site must submit to coordinating center, for example: Knight SAE form, MedWatch form 3500, and/or any other trial specific reporting form (AESI, AECI)].

The Coordinating Center will review and submit SAE information to the FDA, OHSU IRB, sub-sites, and any other entities as required by federal and local policies/regulations, and any other applicable requirements.

The Coordinating Center is responsible for distributing IND and/or IDE Action Letters and/or Safety Reports, as applicable, to sub-sites for review and submission to their institution’s local IRB as required per site policy.

- - 1. FDA Reporting

Some events must be reported to the FDA through the MedWatch Voluntary reporting program, even if the trial involves a commercially available agent. Events to be reported include any UPs (i.e., not listed in the package insert) and any SAEs with a suspected association to the study intervention.

For studies conducted under an IND/IDE, the OHSU Coordinating Center Investigator is required to report certain events to the FDA per applicable regulations.

For multi-site investigator-initiated trials, the OHSU PI is the study sponsor and the following reporting responsibilities and requirements apply.

The sub-site investigator is required to report AEs to the Coordinating Center using a MedWatch report form and/or any trial-specific report form and supporting materials. Adverse events to be reported include any UPs (i.e., not listed in the package insert and/or IB) and any SAEs with a suspected association to the investigational product. The Coordinating Center will centrally assess all reported events and report any events to the FDA as warranted using the MedWatch 3500A Mandatory report form.

- - 1. Suspected Unexpected Adverse Reactions (SUSARs)

Per regulatory requirements, if an event is assessed by the Sponsor Institution as a Serious Unexpected Adverse Reaction (SUSAR), it is the responsibility of the Sponsor Institution to submit the SUSAR to Regulatory Authorities according to applicable regulations. In addition, the SUSAR will be distributed to the Investigators/sites utilizing a Council for International Organizations of Medical Sciences (CIOMS) report form, or the MedWatch 3500A form). The Investigator/site will submit a copy of the report to their respective IRB or IEC per the governing institutional requirements and in compliance with local laws and guidelines.

- - 1. Reporting Of Pregnancy

To ensure participant safety, each pregnancy or suspected pregnancy in a participant during study participation must be reported within 24 hours of learning of its occurrence. The sub-site investigator is required to notify the OHSU Coordinating Center by phone, fax, or email no later than 24 hours of learning of a pregnancy. The sub-site must also report each event to the institution’s local IRB or other oversight entity per institutional policies/requirements. The Coordinating Center will centrally assess the event and report to the manufacturer and any other entity as warranted. The sub-site will send the Coordinating Center information regarding the pregnancy using secure institutional email. Describe what sub-site must submit to Coordinating Center, for example: Knight SAE form template, MedWatch form 3500, and/or any other trial specific reporting form.

The investigator must follow the pregnancy to determine outcome, including spontaneous or voluntary termination, details of the birth, and the presence or absence of any birth defects, congenital abnormalities, or any pregnancy- or childbirth-related and/or newborn complications. The sub-site must report the outcome of the pregnancy to the Coordinating Center as defined above.

If participant becomes pregnant during the study, a separate consent form will be requested and will be submitted via modification. If during study participation a participant’s sexual partner becomes pregnant, the pregnancy and pregnancy outcomes must also be reported as described above. A separate consent form to report information regarding the pregnancy should be obtained from the pregnant individual.

## Study Stopping Rules

The overall study will be paused, and appropriate authorities (e.g., IRB, Knight Data and Safety Monitoring Committee) notified if the following events occur:

• Life-threatening grade 4 toxicity attributable to protocol therapy that is unmanageable, or unexpected.

• Death suspected to be related to ICG injection.

• As indicated by statistical stopping rules in this protocol.

# STATISTICAL CONSIDERATIONS

## Statistical Hypothesis

This is an open label, one arm phase II study to assess the diagnostic perfusion characteristics by intraoperative ICG injection on pancreatic leak rates. It is hypothesized that remnant pancreatic gland perfusion will be readily and objectively evaluated and that hypoperfusion will be associated with increased rates of pancreatic leaks postoperatively.

## Sample Size Determination

This primary objective of study is to detect the difference in leak rates between normal and reduced ICG perfusion group. Since there are no existing data of ICG perfusion in the pancreas, the sample size calculation was not based on the hypothesis but we provided the detectable difference between two groups. Anticipating the balanced number between two groups, our sample size of 50 will have 80% power to detect at least 25% difference in leaks rate between two group assuming 20% leak rate for normal ICG perfusion group.

We consider a liberal exclusion of 30% due to multiple and complex postoperative factors known to be associated with leaks. We anticipate screening 75 patients and enrolling 50.

## Populations for Analyses

11.3.1 Safety population

The safety population includes all enrolled participants who received ICG injection during pancreatectomy. All safety analyses in relation to the intervention will be done with this same population.

11.3.3 Efficacy evaluable population

The efficacy evaluable population includes all participants enrolled in this study who underwent pancreaticoduodenectomy or distal pancreatectomy, received ICG injection and had up to 30 days of postoperative follow-up for complications. Safety analyses for surgery will use the efficacy population.

## Description of Statistical Methods

- - 1. General Approach

Descriptive statistics will be used to summarize patients’ baseline characteristics and baseline disease characteristics. This information will be presented with percentages, means with standard deviations median and range as suitable. Inferential tests will use a null-hypothesis significance testing of <0.05.

- - 1. Analysis of Primary Endpoint(s)

*11.4.2 Primary endpoint – Leak rate*

We will assess the association between ICG perfusion and leaks using a chi-square test. Logistic regression analysis will be further utilized to evaluate covariates influencing the presence/absence of leaks, with specific attention to the predictive value of percent maximum perfusion metrics and time to maximum metrics at the set timepoints in the protocol. Refer to Sections 3.1 for additional information

- - 1. Analysis Of The Secondary Endpoint(S)

*11.4.3.1 Secondary endpoint –*

We will use descriptive statistics to summerize the grade of leasks and ICG metrics.

*11.4.3.2 Secondary endpoint – ICG logistics*

The results of the survey to be applied to surgeons and other OR staff will be summerized using a descriptive statistics.

- - 1. Safety Analyses

Adverse events will be tabulated by the Medical Dictionary for Regulatory Activities (MedDRA Version 21.1) preferred term and system organ class. The severity of the AE will be assessed by National Cancer Institute (NCI) Common Terminology Criteria for Adverse Events (CTCAE) version 5.0. Using the safety analysis set, descriptive statistics will be used to summarize all on-study AEs, grade 3-4 AEs, treatment-related AEs, grade 3-4 treatment-related AEs, SAEs, treatment-related SAEs, and AEs leading to study therapy discontinuation. Grade 3-4 laboratory abnormalities will be summarized using worst grade NCI CTCAE v5.0 criteria.

## Study stopping rules

The overall study will be paused, and appropriate authorities (e.g., IRB, Knight Data and Safety Monitoring Committee) notified if the following events occur:

• Life-threatening grade 4 toxicity attributable to protocol therapy that is unmanageable, or unexpected.

• Death suspected to be related to ICG injection.

• As indicated by statistical stopping rules in this protocol.

- - 1. Baseline Descriptive Statistics

Summaries of patient demographics, baseline characteristics and baseline disease characteristics will be presented for participants using the safety evaluable population to include the following:

1. Demographics: Sex; Age; Ethnicity; Race; Region of subsite
2. Baseline characteristics: Height (cm); Weight (kg); ECOG performance status
3. Baseline disease characteristics: Disease stage at study entry; cancer therapy; biomarker status

## Handling of Missing Data

Patient and intervention data will be carefully recorded. In the event that a specific data point could not be recorded, no input or analysis will be done with missing data. Missing data will be reported as such and if esencial information is not found, participant may be excluded entirely or partially from the study.

# CLINICAL MONITORING

## OHSU Knight Cancer Institute Data & Safety Monitoring Plan

All clinical trials at the Knight are required to have Data and Safety Monitoring Plan (DSMP). This study is under the oversight of the Knight Cancer Institute’s DSMC as described in the Knight institutional DSMP. The Knight DSMP outlines the elements required to ensure the safety of clinical trial participants, the accuracy and integrity of the data, and the appropriate modification of cancer-related clinical trials for which significant benefits or risks have been discovered or when the clinical trial cannot be successfully concluded. The Knight DSMP also describes the methods and procedures for ensuring adequate, risk-based oversight of cancer-related research at OHSU.

As described in the Knight DSMP, regardless of a trial’s risk level and any specific Knight oversight in place, the Investigator is singularly responsible for overseeing every aspect of the design, conduct, and final analysis of his/her investigation.

The Knight DSMC reviews and monitors study progress, toxicity, safety, and other data for this study. The DSMC will address any issue that raises questions about data integrity or trial participant safety with the Investigator and study team. Should any major concern arise, the Knight DSMC may recommend corrective action, and determine whether to suspend or terminate the study.

## Clinical Data & Safety Monitoring

As part of the Quality Assurance plan and in full agreement with NIH policy (NIH Guide, NIH Policy for Data and Safety Monitoring, June 10, 1998) that states all clinical trials require monitoring to ensure the safety of study participants and the validity and integrity of the data, monitoring will be a continuous, ongoing and multifaceted process. This includes external review by the DSMC and IRB(s), as well as internal data quality control, review and evaluation. Site monitoring visits are central to this process, and will include reporting to appropriate individuals with oversight responsibilities.

The OHSU Investigator is ultimately, singularly responsible for overseeing every aspect of the investigation, including design, governing conduct at all sub-sites, and final analysis of study data.

In the absence of a formal monitoring plan, the Investigator may work with his/her study team to conduct and document internal monitoring of the study to verify protection of human participants, quality of data, and/or ongoing compliance with the protocol and applicable regulatory requirements.

If at any time Investigator noncompliance is discovered at OHSU or any sub-site, the Investigator shall promptly either secure compliance or end the Investigator’s participation in the study.

Independent audits will be conducted by the Knight DSMC to verify that the rights and well-being of human participants are protected, that the reported trial data are accurate, that the conduct of the trial is in compliance with the protocol and applicable regulatory requirements, and that evidence of ongoing investigator oversight is present.

## Quality Assurance & Quality Control

The investigational site will provide direct access to all trial-related source data/documents and reports for monitoring by the monitor and/or sponsor, and auditing by the Knight DSMC and/or regulatory authorities.

All Knight Cancer Institute clinical trials must have a Data and Safety Monitoring Plan (DSMP). All clinical work conducted under this protocol is subject to ICH GCP guidelines. This includes inspection of study-related records by the lead site, Sponsor, its designee, or health authority representatives at any time.

QA audit activities will occur as detailed in the Knight’s institutional DSMP. All discrepancies, queries, deviations, observations, and findings of non-compliance will be compiled into a final audit report. The PI must review and assess each finding and generate a response to the audit report that incorporates Corrective and Preventative Action (CAPA). A CAPA must approach analyzing root cause(s) of noncompliance in order to identify and determine changes to correct and resolve issues and prevent recurrence.

Quality Control (QC) activities will occur to monitor and ensure the safety of study participants and the validity and integrity of data. Monitoring will be a continuous, ongoing, and multifaceted process. This includes review by the Knight DSMC and applicable IRB(s), as well as internal data quality control, review and evaluation. Site monitoring visits are central to this process and will include reporting to appropriate individuals with oversight responsibilities.

The Sponsor-Investigator, or study monitor, will verify that the clinical trial is conducted, and data are generated, documented (recorded), and reported in compliance with the protocol, GCP, and the applicable regulatory requirements (e.g., Good Laboratory Practices (GLP), Good Manufacturing Practices (GMP)).

# SUPPORTING DOCUMENTATION AND OPERATIONAL CONSIDERATIONS

## Source Data/Documents

The Investigator is responsible for ensuring the accuracy, completeness, legibility, and timeliness of the data reported. All source documents should be completed in a neat, legible manner to ensure accurate interpretation of data. The Investigator will maintain adequate case histories of study participants, including accurate CRFs, electronic (e)CRFs and relevant electronic data capture (EDC) system (if applicable), and all relevant source documentation.

- - 1. Participant & Data Confidentiality

The information obtained during this clinical study is confidential, and unless otherwise noted, disclosure to third parties is prohibited. Information contained within this study will be maintained in accordance with applicable laws protecting participant privacy, including the provisions of the Health Insurance Portability and Accountability Act (HIPAA).

Participant confidentiality is strictly held in trust by the site Investigator(s) and study team. This confidentiality is extended to cover testing of biological samples and genetic tests and the clinical information about participants. Therefore, the study protocol, documentation, data, and all other information generated will be held in strict confidence. No information concerning the study, or the data will be released to any unauthorized third party without prior written approval of the sponsor.

The study monitor, other authorized representatives of the sponsor, representatives of the IRB or manufacturer supplying study product may inspect all documents and records required to be maintained by the Investigator, including but not limited to, medical records (office, clinic, or hospital) and pharmacy records for the participants in this study. The clinical study site will permit access to such records.

The study participant’s contact information will be securely stored at each clinical site for internal use during the study. At the end of the study, all records will continue to be kept in a secure location for as long a period as dictated by local IRB and institutional regulations. Study participant research data, which is for purposes of statistical analysis and scientific reporting, will be transmitted to and stored within the Knight Cancer Institute per [OHSU’s Information Security Directives](http://www.ohsu.edu/xd/about/services/integrity/policies/ips-policies-by-category.cfm). Individual participants and their research data will be identified by a unique study identification number. The study data entry and study management systems used by clinical sites and by Knight Cancer Institute research staff will be secured and password protected per [OHSU’s Information Security Directives](http://www.ohsu.edu/xd/about/services/integrity/policies/ips-policies-by-category.cfm). At the end of the study, or after the appropriate period of record retention stated in Section 13.1.4, all study databases will be de-identified and archived within the Knight Cancer Institute.

- - 1. Data Collection & Storage: Privacy, Confidentiality & Security

Data collection is the responsibility of the clinical trial staff at the site under the supervision of the site Investigator. The Investigator is responsible for ensuring the accuracy, completeness, legibility, and timeliness of the data reported. Standard institutional practices will be followed as described in the [OHSU’s Information Security Directives](http://www.ohsu.edu/xd/about/services/integrity/policies/ips-policies-by-category.cfm) to maintain the confidentiality and security of data collected in this study. Study staff will be trained on these procedures.

Loss of participant confidentiality is a risk of participation. Efforts will be made to keep study participant identities confidential except as required by law. Participants’ samples will be identified by code only. Specifically, each consenting participant will be assigned a unique coded identifier consisting of numbers. This identifier will be associated with the participant throughout their participation in the trial. The coded identifier will also be used to identify any participant specific samples.

Basic accrual tracking information (demographic, consent, visit information) will be captured in OHSU’s electronic clinical research information system (eCRIS), hosted on OHSU secure servers, and managed by OHSU’s information technology group at their data center in downtown Portland, Oregon. Any additional printed documents containing participant identifiers, such as those from the medical record to confirm eligibility, will be filed in binders and kept in a locked, secure location.

Study outcome data will be captured in electronic case report forms (eCRFs) using an electronic data capture (EDC) system approved by OHSU’s office of Information Privacy and Security. To preserve confidentiality, PHI in the EDC system will be limited to just birth date and visit dates. The web-accessible EDC system is password protected and encrypted with role-based security and administered by designated informatics staff within OHSU or Knight Cancer Institute. All users of the database are assigned a unique ID, username, and password and must complete training appropriate to their role before they are authorized to enter, access, and store data in the database.

Where applicable, data from correlative studies may be entered into the EDC system by study personnel. All other electronic data extracts will be stored only on local study site computers and restricted drives, which are limited to only study investigators and staff with authorization to access the data. Quality assurance will be conducted as outlined in Section 12.3, Quality Assurance & Quality Control

*13.1.3 National Surgical Quality Improvement Project (NSQIP) & Efficacy Data Collection*

NSQIP is an international initiative sponsored by the American College of Surgeons that records patient demographics, treatment regarding surgical interventions, and captures 30-day postoperative complications data. “Procedure-targeting” allows institutions to capture 100% of certain surgical procedures along with an expanded set of procedure-specific datapoints. OHSU has been procedure-targeting pancreatectomies for a decade.

Data collection for 30-day postoperative complications, including rates of fistula formation, will be performed by a trained NSQIP surgical clinical reviewer. Due to the workflow of NSQIP reviewers, a lag period between surgery is anticipated equaling up to 3-months. For the interim efficacy analysis, subject charts may be reviewed for information relevant to the primary endpoint by individuals other than the NSQIP reviewer. Data obtained from the NSQIP reviewer relating to primary or secondary endpoints will be separately reviewed by a member of the study team and any conflicts or discrepancies resolved. Finalized data will be entered into the EDC system and used for final analysis.

- - 1. Future Use of Stored Specimens and Data

With patient’s and IRB approval, tissue samples from the pancreas will be stored within the Oregon Pancreas Tissue Registry IRB00003609. Consent for aforementioned IRB is separate from this study (25055). Collection of blood could also be performed for further studies. These samples are used to better understand pancreatic pathologies and their complications. Consent of tissue storage can be withdrawn at any time by patients.

- - 1. Maintenance of Records

Records and documents pertaining to the conduct of this study, source documents, consent forms, laboratory test results and medication inventory records, must be retained by the Investigator for a period of 2 years following the date a marketing application is approved for the drug for the indication for which it is being investigated; or, if no application is to be filed or if the application is not approved for such indicate, until 2 years after the investigation is discontinued and FDA is notified. It is the responsibility of the sponsor to inform the Investigator when these documents no longer need to be retained.

If the Investigator relocates or for any reason withdraws from the study, the study records must be transferred to an agreed upon designee, such as another institution or another investigator at OHSU. Records must be maintained according to institutional or FDA requirements.

## Multi-Site Guidelines

Each sub-site is expected to maintain appropriate medical and research records in compliance with ICH GCP and regulatory and institutional requirements for the protection and confidentiality of participants.

OHSU Coordinating Center will provide a manual of procedures and/or data management plan to sub-sites to describe required data and document submission requirements and timelines.

OHSU Coordinating Center will communicate with sub-site(s) on a routine basis and will retain essential documents in accordance with ICH GCP and regulatory and institutional requirements.

## Publication and Data Sharing Policy

This study will be conducted in accordance with the following publication and data sharing policies and regulations:

National Institutes of Health (NIH) Public Access Policy, which ensures that the public has access to the published results of NIH funded research. It requires scientists to submit final peer-reviewed journal manuscripts that arise from NIH funds to the digital archive PubMed Central upon acceptance for publication.

This study will comply with the NIH Data Sharing Policy and Policy on the Dissemination of NIH-Funded Clinical Trial Information and the Clinical Trials Registration and Results Information Submission rule. As such, this trial will be registered at ClinicalTrials.gov, and results information from this trial will be submitted to ClinicalTrials.gov. In addition, every attempt will be made to publish results in peer-reviewed journals. Data from this study may be requested from other researchers 2 years after the completion of the primary endpoint by contacting the PI.

This study will comply with the NIH Public Access Policy, which ensures that the public has access to the published results of NIH funded research. It requires scientists to submit final peer-reviewed journal manuscripts that arise from NIH funds to the digital archive PubMed Central upon acceptance for publication.

This study will adhere to the requirements set forth by the ICMJE and FDAAA that requires all clinical trials to be registered in a public trials registry (e.g., ClinicalTrials.gov) prior to participant enrollment.

## Conflict of Interest Policy

The independence of this study from any actual or perceived influence, such as by the pharmaceutical industry, is critical. Therefore, any actual conflict of interest of persons who have a role in the design, conduct, analysis, publication, or any aspect of this trial will be disclosed and managed. Furthermore, persons who have a perceived conflict of interest will be required to have such conflicts managed in a way that is appropriate to their participation in the design and conduct of this trial. Conflicts of interest, for all study group members, should be disclosed and managed according to OHSU’s established policies and procedures.

Refer to link: <https://o2.ohsu.edu/integrity-department/conflict-of-interest/index.cfm>

# ETHICS/PROTECTION OF HUMAN PARTICIPANTS

## Ethical Standard

The Investigator will ensure that this study is conducted in full conformity with Regulations for the Protection of Human Participants of Research codified in 45 CFR Part 46, 21 CFR Part 50, 21 CFR Part 56, and/or the ICH E6.

## Institutional Review Board

The protocol, informed consent form(s), recruitment materials, and all participant materials will be submitted to the IRB for review and approval. Approval of both the protocol and the consent form must be obtained before any participant is enrolled. Any amendment to the protocol will require review and approval by the IRB before the changes are implemented to the study. All changes to the consent form will be IRB approved; a determination will be made regarding whether previously consented participants need to be re-consented.

## Informed Consent

Written informed consent will be obtained from all participants participating in this trial, as stated in the Informed Consent section of [21 CFR Part 50](https://www.accessdata.fda.gov/scripts/cdrh/cfdocs/cfcfr/CFRSearch.cfm?CFRPart=50). Documentation of the consent process and a copy of the signed consent shall be maintained in the participant’s medical record.

- - 1. Consent Procedures and Documentation

Informed consent is a process that is initiated prior to the individual’s agreement to participate in the study and continues throughout the individual’s study participation. Extensive discussion of risks and possible benefits of participation will be provided to the participants and their families as appropriate. Consent forms will be IRB-approved, and the participant will be asked to read and review the document. The Investigator will explain the research study to the participant and answer any questions that may arise. All participants will receive a verbal explanation in terms suited to their comprehension of the purposes, procedures, and potential risks/benefits of the study, alternatives to participation, and of their rights as research participants. Participants will have the opportunity to carefully review the written consent form and ask questions prior to signing. The participants should have the opportunity to discuss the study with their surrogates or think about it prior to agreeing to participate. The participant will sign the informed consent document prior to any procedures being done specifically for the study. The participants may withdraw their consent at any time throughout the course of the trial. A copy of the informed consent document will be given to the participants for their records. The rights and welfare of the participants will be protected by emphasizing to them that the quality of their medical care will not be adversely affected if they decline to participate in this study.

## Protocol Review

The protocol and informed consent form for this study must be reviewed and approved in writing by the OHSU Knight Cancer Institute’s Clinical Research Review Committee (CRRC) and the appropriate IRB prior to any participant being consented on this study. All study sites must have IRB approval of protocol by IRB of record before consenting any participants.

## Changes to Protocol

Any modification of this protocol must be documented in the form of a protocol revision or amendment submitted by the Investigator and approved by the CRRC and IRB, before the revision or amendment may be implemented. The only circumstance in which the amendment may be initiated without regulatory approval is for a change necessary to eliminate an apparent and immediate hazard to the participant. In that event, the Investigator must notify the IRB (and sponsor/FDA if under an IND/IDE) within 5 business days after the implementation.

# REFERENCES

1. American Cancer Society. Cancer Statistics Center. 2023 [Cited 05/07/23]. Available from: <http://cancerstatisticscenter.cancer.org>
2. Strobel O, Lorenz P, Hinz U, et al. Actual Five-year Survival After Upfront Resection for Pancreatic Ductal Adenocarcinoma: Who Beats the Odds? Ann Surg. 2022 May 1;275(5):962-971. doi: 10.1097/SLA.0000000000004147. Epub 2020 Jul 7. PMID: 32649469.
3. Merkow RP, Bilimoria KY, Tomlinson JS, et al. Postoperative Complications Reduce Adjuvant Chemotherapy Use in Resectable Pancreatic Cancer: Ann Surg 2014;260:372–7. https://doi.org/10.1097/SLA.0000000000000378.
4. Maeda K, Kuriyama N, Yuge T, et al. Risk factor analysis of postoperative pancreatic fistula after distal pancreatectomy, with a focus on pancreas-visceral fat CT value ratio and serrated pancreatic contour. BMC Surg. 2022 Jun 22;22(1):240. doi: 10.1186/s12893-022-01650-8. PMID: 35733145; PMCID: PMC9215066.
5. Zhou Q, He W, Liu Y, et al. Drainage volume on postoperative day one to predict clinically relevant postoperative pancreatic fistula following distal pancreatectomy. BMC Surg. 2022 Aug 1;22(1):297. doi: 10.1186/s12893-022-01748-z. PMID: 35909183; PMCID: PMC9341036.
6. Macchi V, Picardi EEE, Porzionato A, Morra A, Bardini R, Loukas M, Tubbs RS, De Caro R. Anatomo-radiological patterns of pancreatic vascularization, with surgical implications: Clinical and anatomical study. Clin Anat. 2017 Jul;30(5):614-624. doi: 10.1002/ca.22885. Epub 2017 May 10. PMID: 28395109.
7. Bertelli E, Di Gregorio F, Bertelli L, Orazioli D, Bastianini A. The arterial blood supply of the pancreas: a review. IV. The anterior inferior and posterior pancreaticoduodenal aa., and minor sources of blood supply for the head of the pancreas. An anatomical review and radiologic study. Surg Radiol Anat. 1997;19(4):203-12. PMID: 9381324.
8. Strasberg SM, McNevin MS. Results of a technique of pancreaticojejunostomy that optimizes blood supply to the pancreas. J Am Coll Surg 1998;187:591–6. https://doi.org/10.1016/S1072-7515(98)00243-9.
9. Connor S. Defining post-operative pancreatitis as a new pancreatic specific complication following pancreatic resection. HPB 2016;18:642–51. https://doi.org/10.1016/j.hpb.2016.05.006.
10. Nahm CB, Brown KM, Townend PJ, et al. Acinar cell density at the pancreatic resection margin is associated with post-pancreatectomy pancreatitis and the development of postoperative pancreatic fistula. HPB 2018;20:432–40. https://doi.org/10.1016/j.hpb.2017.11.003.
11. Martin AN, Narayanan S, Turrentine FE, et al. Pancreatic duct size and gland texture are associated with pancreatic fistula after pancreaticoduodenectomy but not after distal pancreatectomy. PLoS One. 2018 Sep 13;13(9):e0203841. doi: 10.1371/journal.pone.0203841. PMID: 30212577; PMCID: PMC6136772.
12. Casadei R, Ricci C, Taffurelli G, et al. Prospective validation of a preoperative risk score model based on pancreatic texture to predict postoperative pancreatic fistula after pancreaticoduodenectomy. Int J Surg. 2017 Dec;48:189-194. doi: 10.1016/j.ijsu.2017.09.070. Epub 2017 Oct 5. PMID: 28987563.
13. Takagi K, Umeda Y, Yoshida R, et al. Robotic spleen-preserving distal pancreatectomy using indocyanine green fluorescence imaging (with video). Asian J Surg. 2022 Jan;45(1):596-597. doi: 10.1016/j.asjsur.2021.10.005. Epub 2021 Nov 17. PMID: 34801368.
14. Saito K, Morimoto M, Denda Y, et al. Accurate intraoperative real-time blood flow assessment of the remnant stomach during robot-assisted distal pancreatectomy with celiac axis resection using indocyanine green fluorescence imaging and da Vinci Firefly technology. Asian J Endosc Surg. 2023 Jan 30. doi: 10.1111/ases.13169. Epub ahead of print. PMID: 36718050.
15. Rho SY, Kim SH, Kang CM, Lee WJ. Is ICG-enhanced image able to help predicting pancreatic fistula in laparoscopic pancreaticoduodenectomy? Minim Invasive Ther Allied Technol. 2019 Feb;28(1):29-32. doi: 10.1080/13645706.2018.1479271. Epub 2018 Jun 5. PMID: 29869569.
16. Park W, Chawla A, O'Reilly EM. Pancreatic Cancer: A Review. JAMA. 2021 Sep 7;326(9):851-862. doi: 10.1001/jama.2021.13027. Erratum in: JAMA. 2021 Nov 23;326(20):2081. PMID: 34547082; PMCID: PMC9363152.
17. Goh BK, Tan YM, Chung YF, Cheow PC, Ong HS, Chan WH, Chow PK, Soo KC, Wong WK, Ooi LL. Critical appraisal of 232 consecutive distal pancreatectomies with emphasis on risk factors, outcome, and management of the postoperative pancreatic fistula: a 21-year experience at a single institution. Arch Surg. 2008 Oct;143(10):956-65. doi: 10.1001/archsurg.143.10.956. PMID: 18936374.
18. Birkmeyer JD, Siewers AE, Finlayson EV, Stukel TA, Lucas FL, Batista I, Welch HG, Wennberg DE. Hospital volume and surgical mortality in the United States. N Engl J Med. 2002 Apr 11;346(15):1128-37. doi: 10.1056/NEJMsa012337. PMID: 11948273.
19. Wheeler HO, CRANSTON WI, MELTZER JI. Hepatic uptake and biliary excretion of indocyanine green in the dog. Proc Soc Exp Biol Med. 1958 Oct;99(1):11-4. doi: 10.3181/00379727-99-24229. PMID: 13601749.
20. Asbun D, Kunzler F, Marin R, Asbun HJ. Pancreatic fluorescence using continuous indocyanine green infusion. J Surg Oncol. 2022 Dec;126(7):1215-1218. doi: 10.1002/jso.27055. Epub 2022 Aug 9. PMID: 35943342.
21. Rho SY, Kim SH, Kang CM, Lee WJ. Is ICG-enhanced image able to help predicting pancreatic fistula in laparoscopic pancreaticoduodenectomy? Minim Invasive Ther Allied Technol. 2019 Feb;28(1):29-32. doi: 10.1080/13645706.2018.1479271. Epub 2018 Jun 5. PMID: 29869569.
22. de Muynck LDAN, White KP, Alseidi A, et al. Consensus Statement on the Use of Near-Infrared Fluorescence Imaging during Pancreatic Cancer Surgery Based on a Delphi Study: Surgeons' Perspectives on Current Use and Future Recommendations. Cancers (Basel). 2023 Jan 20;15(3):652. doi: 10.3390/cancers15030652. PMID: 36765609; PMCID: PMC9913161.
23. Kwiterovich KA, Maguire MG, Murphy RP, Schachat AP, Bressler NM, Bressler SB, Fine SL. Frequency of adverse systemic reactions after fluorescein angiography. Results of a prospective study. Ophthalmology. 1991 Jul;98(7):1139-42. doi: 10.1016/s0161-6420(91)32165-1. PMID: 1891225.
24. Hope-Ross M, Yannuzzi LA, Gragoudas ES, Guyer DR, Slakter JS, Sorenson JA, Krupsky S, Orlock DA, Puliafito CA. Adverse reactions due to indocyanine green. Ophthalmology. 1994 Mar;101(3):529-33. doi: 10.1016/s0161-6420(94)31303-0. PMID: 8127574.
25. Kim M, Lee S, Park JC, Jang DM, Ha SI, Kim JU, Ahn JS, Park W. Anaphylactic Shock After Indocyanine Green Video Angiography During Cerebrovascular Surgery. World Neurosurg. 2020 Jan;133:74-79. doi: 10.1016/j.wneu.2019.09.135. Epub 2019 Sep 28. PMID: 31574334.
26. Dip F, Lo Menzo E, Bouvet M, Schols RM, Sherwinter D, Wexner SD, White KP, Rosenthal RJ. Intraoperative fluorescence imaging in different surgical fields: Consensus among 140 intercontinental experts. Surgery. 2022 Dec;172(6S):S54-S59. doi: 10.1016/j.surg.2022.07.025. PMID: 36427931.
27. Cassinotti E, Al-Taher M, Antoniou SA, Arezzo A, et al. European Association for Endoscopic Surgery (EAES) consensus on Indocyanine Green (ICG) fluorescence-guided surgery. Surg Endosc. 2023 Mar;37(3):1629-1648. doi: 10.1007/s00464-023-09928-5. Epub 2023 Feb 13. PMID: 36781468; PMCID: PMC10017637.

1. Bassi C, Marchegiani G, Dervenis C, Sarr M, Abu Hilal ; International Study Group on Pancreatic Surgery (ISGPS). The 2016 update of the International Study Group (ISGPS) definition and grading of postoperative pancreatic fistula: 11 Years After. Surgery. 2017 Mar;161(3):584-591. doi: 10.1016/j.surg.2016.11.014. Epub 2016 Dec 28. PMID: 28040257.
