# Supplementary material for: The VIPR-1 trial (Visualizing Ischemia in the Pancreatic Remnant): Assessing the role of intraoperative indocyanine green perfusion in predicting postoperative pancreatic leaks and fistulas: Protocol for a phase II clinical trial
Source: PLoS One. 2025 Jun 24;20(6):e0311025. doi: 10.1371/journal.pone.0311025 (PMC12186879; doi:10.1371/journal.pone.0311025)
Supplement: Attachment — Submitted filename: Response to Reviewers.docx [file pone.0311025.s005.docx]

**Journal Requirements**

1. We have changed the file names to match those required for PLOS ONE style.
2. We have removed the institutional logo from the Original Protocol for copyright reasons.
3. We have updated the cover letter to reflect the Role of Funder statement.
4. The corresponding author’s ORCID is now registered in Editorial Manger.
5. The titles in Editorial Manager and the manuscript file now match.
6. Ethical statement under the methods section now includes the full name of the IRB and ethics committee that oversees the approval of the clinical trial at our institution.
7. References were checked and completed.

**Reviewer 1**:

Dear reviewer, thank you for taking the time to go over our protocol.

1. No revisions suggested.

**Reviewer 2**:

Thank you for your insights and comments regarding our clinical trial protocol, please find our responses below.

1. We believe that specifying criteria for pancreatectomy after trauma is something that we had left out. Because of this, we now specify in our first inclusion criteria that only elective pancreatectomies are included. These changes can be seen in Page 5, line 106 of the manuscript. We have amended this in our IRB’s entry for the trial protocol as well.
2. The reason behind principal surgeon blinding is to eliminate surgical margin decision intraoperatively based on the metrics captured. The secondary surgeon / first assistant stays scrubbed during the image capturing period of the case. This is done because of institutional guidelines where at least one surgeon needs to stay scrubbed in during the imaging portion of the case. However, all objective metrics (protocol document lines 167-170) are calculated with the imaging platform physically outside of the operating room to avoid impacting any surgical and postoperative treatment decisions. For example, a surgeon or resident might be inclined to pursue a prophylactic measure such as a somatostatin analog to reduce postoperative fistula if they saw that a participant had objective measurements hinting hypoperfusion of the pancreas. Because of this, after the images are captured, the primary surgeon then can re-enter the surgical field and continue with the surgery.

**Reviewer 3**:

We greatly appreciate your time and efforts while reviewing our manuscript. Please find our point-by-point responses directly below.

1. We have condensed our introduction to 4 paragraphs by deleting redundant sentences. We hope this leads to an easier read while still highlighting the importance of this study. These changes are seen in lines 63-65, 70-74 and 76-79 in the tracked changes document.
2. Line 75 has been edited to remove the statement mentioning a future development of intraoperative interventions because this would be a part of a separate study.
3. Levels for categorical variables for covariates captured are now specified. Type of pancreatectomy whether proximal versus distal, and gland texture – soft, intermediate or firm. (Line 187 and 189).
