# Supplementary material for: The VIPR-1 trial (Visualizing Ischemia in the Pancreatic Remnant): Assessing the role of intraoperative indocyanine green perfusion in predicting postoperative pancreatic leaks and fistulas: Protocol for a phase II clinical trial
Source: PLoS One. 2025 Jun 24;20(6):e0311025. doi: 10.1371/journal.pone.0311025 (PMC12186879; doi:10.1371/journal.pone.0311025)
Supplement: Attachment — Submitted filename: Response to Reviewers R2.docx [file pone.0311025.s006.docx]

Reviewer #1:

I appreciate your revisions incorporated as suggested by my worthy reviewer colleague. It’s now more appropriate.

**Response**: Reviewer 1, thank you for your valuable feedback.

Reviewer #2:

I am thankful to the authors for their responses.
I would like to have some queries answered by the authors.

1. This protocol is to assess the role of intraoperative ICG perfusion of transected pancreas in predicting leaks and or fistula. however, the primary outcome mentioned is the correlation between ICG measurements and the development or absence of fistula formation. As per International Study Group in Pancreatic Surgery (ISGPS), previously Grade-A fistula is no longer defined as fistula. So, I would like clarity on whether authors are going including biochemical leaks in the study. That has to be clearly mentioned in the title as well as in the text.

**Response**: Reviewer #2, you raise a very important point regarding the current classification of fistulas and leaks. We intend to evaluate the primary outcome of both fistulas and biochemical leaks in our trial. We have reworded the title and main outcomes to better reflect this. We also intend to conduct a subgroup analysis regarding the predictive potential of ICG measurements for all grade fistulas/leaks, A, B, and C and only clinically relevant, B-C fistulas. The modifications to the manuscript can be seen in lines 1-3, 33, and 176 of the tracked changes document.

2. Oppermann C et al in 2023 noticed that there was a large variation in fluorescence intensity between different organs and between the same organ in different subjects while using a fixed weight-adjusted dosing regimen using the same camera setting and placement (in piglet models). How can this variation be minimized and what would be its effects on the expected outcomes as the hypoperfusion of pancreas would be in relation to other structures in this study.

**Response**: the works from Opperman et al. were of paramount importance for our study protocol design and we cited it since the first iteration of our submission. Notably, we standardized the camera-organ distance with the built-in function of visible red lasers in the SpyElite platform to diminish variations between one participant and another. These two lasers converge at an optimal distance from the organ and then the measurement is performed. The SpyElite camera hovers above the patient fixed with an arm attached to the main body of the device. This allows for stable and repeatable measurements between participants. In contrast Opperman opted to use the Stryker 1588 which is a hand-held camera, which could be more prone to inter-user variations regarding camera-organ distances. Furthermore, Opperman reports that Tmax and TR–time ratio (T1/2 max/Tmax) appear as the most stable metrics between a piglet model and the next one. We are able to capture these two metrics in our current workflow. Additionally, the camera used by Opperman needs to be switched between visible light and near infrared light, due to the laparoscopic nature of the camera. On the other hand, the SpyElite camera only captures near infrared light when recording. We acknowledge the limitations that could be encountered between participants and hope to report these same variations when we publish our results. We believe that tissue perfusion variation between participants can be accounted for by measuring the difference or delta within the same participant regarding a reference tissue and the target tissue (pancreas). However, the trial itself would explore the impact of these variations to the predictive capabilities of tissue perfusion with ICG. This is further adjusted for with our greater number of planned participants (N = 50) compared to Opperman’s proof of concept study with a porcine model (N = 3).

3. Line 165, "The main outcome will be the development or absence of postoperative pancreatic fistula", however the patients with SBP<90 mmHg, ICU transfer, Blood Transfusion and vasopressor support between postoperative days 3-5 are to be excluded from the study. What should be the standard post-operative time after which a complication could be attributed to pancreatic fistula because all the above-mentioned complications can well happen because of pancreatic fistula. Kindly provide the rationale for excluding these patients from the study.

**Response:** The reviewer makes an excellent point and our criteria were defined in order to best select out complications from immediate technical errors that could arise postoperatively that might cloud the analysis of pancreatic hypoperfusion intraoperatively (e.g., postoperative bleeding, sepsis, organ failure), or that may subsequently increase the risk of a crPOPF. While it is true that crPOPF can occur in this timeframe and cause these same consequences, 1) this has not been observed in screened patients and 2) critical illness resulting from a crPOPF typically to present with later consequences, which is why we went no further than POD5. Our goal was to attempt to select for leaks that resulted from ischemia detectable intraoperatively and not from other potential causes of hypoperfusion that are not potentially modifiable intraoperatively. It is challenging to define a standard postoperative interval during which a complication could be attributed to a fistula, but as it is common practice to test drain amylase on postoperative days 3-4, we felt excluding immediate postoperative complications after a presumed period of adequate resuscitation would be appropriate.
4. Line-142. "Still pictures will be extracted from the video at 10-second intervals once tissue saturation is stable". For how long will these images be taken?

**Response**: We have added the minimal duration of capturing ICG metrics with video recording within the SpyElite platform in line 153-154.

5. Line-167 "we will collect surgical drain amylase levels in the first three days after surgery,..) needs to be addressed as POPF as per original ISGPS definition was diagnosed when the amylase content was greater than 3 times the upper normal serum value starting from the postoperative day 3 (rather than first 3 days after surgery). The pancreatic fistula definition per se is unchanged, the criteria for its diagnosis underwent a change.

**Response**: Thank you for this comment, we have corrected these lines of text in the manuscript to better reflect what is shown in the Table for Schedule of Procedures and Evaluations in the original protocol. Drain amylase is captured at least once during day 3 postop and any subsequent measurements would depend on patient specific scenarios (serial measurements after a high amylase is detected, until drain is removed). These changes can be seen in lines 178-182 from the manuscript with tracked changes enabled.

Reviewer #3:

I believe my comments have been addressed appropriately. I would like to applaud the authors again for a well written manuscript.

**Response**: Reviewer 3, thank you for your valuable feedback.

Reviewer #4: 
The study’s key measurements:
Exposure:
ICG measurements of the cut edge of the pancreas will be recorded before reconstruction.

Endpoint:
International Study Group on Pancreatic Surgery criteria for pancreatic fistula will be used to
define leaks and fistulas.

Then the protocol says:

“The primary outcome will be the correlation between ICG measurements and the development or absence of fistula formation.”

This is not the primary outcome, rather it is the study objective: to assess potential association of ICG measurements (X variable, so to speak) and the development or absence of fistula formation (dependent variable Y).

**Response**: thank you for your correction. We have made this change, rewording this line to reflect that this is our study objective rather than our primary outcome. These changes can be seen in lines 32-34 of the manuscript with tracked changes.

Potential covariates: conventional measurements. What are those?

**Response**: we have specified potential covariates and conventional measurements in lines 77-79. Covariates are listed in lines 190-194 of the manuscript with tracked changes.

Sample size calculation needs to be specified:

Anticipating balanced numbers between the two groups, our sample size of 50 participants will have power to detect at least a 25% difference in a leak or fistula rate, assuming a 20% leak rate for the normal ICG perfusion group. We account for a liberal exclusion of 30% of participants due to multiple and complex postoperative factors known to be associated with leaks. Therefore, we anticipate screening 75 participants and enrolling 50.

Do you expect equal number of subjects in the fistula and no fistula groups? Or are you going to control them to be equal?
If so, what’s the difference between them on the ICG measurements?

Or are you going to build a predictive model using ICG measurements to predict the fistula and no fistula status post-surgery as you stated earlier? This requires difference sample size justification.

**Response:** Imbalance Between Groups:

We do not expect equal numbers of subjects in the fistula and no-fistula groups. Given that approximately 20% of patients in the normal perfusion group are expected to develop a fistula, while 50% of patients in the poor perfusion group are expected to develop one, there will naturally be a greater proportion of non-fistula patients in the normal perfusion group. Based on this, we have calculated the sample sizes for the two groups as follows:

Normal perfusion group (n1): 40 participants

Poor perfusion group (n2): 10 participants

Total sample size: 50 participants

Statistical Justification:

The imbalance ratio between the groups (approximately 4:1) is accounted for in our sample size calculation. The required sample size was determined to achieve 80% power and 5% significance level for detecting a meaningful difference in fistula rates between the groups. We used an effect size calculation based on the difference in expected proportions of fistulas (20% vs. 50%) between the two groups. Changes in the manuscript can be seen in lines 93-103 of the tracked changes file.

Predictive Model:

While we plan to use ICG measurements to predict fistula development, the primary focus of this study is to assess the impact of ICG perfusion on fistula formation post-surgery, with statistical power focused on comparing the proportions of fistulas between the normal and poor perfusion groups. If predictive modeling is pursued in a follow-up analysis, a separate sample size calculation will be performed to determine the appropriate number of participants needed for model training and validation, as this would involve different statistical considerations, including potential adjustments for covariates.

We hope this clarifies our approach to sample size and group imbalance. Please let us know if further elaboration is needed. We hypothesize that ICG can be used in conjunction with other known risk factors to further improve postoperative pancreatic fistula risk prediction.
