# Supplementary material for: The VIPR-1 trial (Visualizing Ischemia in the Pancreatic Remnant): Assessing the role of intraoperative indocyanine green perfusion in predicting postoperative pancreatic leaks and fistulas: Protocol for a phase II clinical trial
Source: PLoS One. 2025 Jun 24;20(6):e0311025. doi: 10.1371/journal.pone.0311025 (PMC12186879; doi:10.1371/journal.pone.0311025)
Supplement: Attachment — Submitted filename: Response to Reviewers R3.docx [file pone.0311025.s007.docx]

Reviewer #2:

Thank you for handling the queries and updating the manuscript. The manuscript is more appropriate now.

I suggest minor polishing of lines 153-154 of tracked changes document.

**Response from authors**: Dear Reviewer, we have now improved the wording of the sentence you pointed out. Thank you for your comments. These changes are in the tracked manuscript and in the latest revised version.

Reviewer #3:

The protocol states:

“We anticipate a 20% incidence of fistula in the normal perfusion group, and at least a 50% incidence in the poor perfusion group is expected. With an expected imbalance ratio of approximately 4:1 between these groups, a total sample size of 50 participants (40 in the normal perfusion group and 10 in the poor perfusion group) provides 80% power at a 5% significance level to detect this difference.”

My calculations show that:

40 in the normal perfusion group and 10 in the poor perfusion group with expected 20% and 50% incidence of fistula, the study has 49.1% power using chi-squared test and 35% power using exact test.

Could you explain how did you get 80% power?

**Response from authors:**

Dear Reviewer,

Thank you for your meticulous review and for identifying the inconsistency in our power calculation. We sincerely appreciate your diligence. Below, we clarify the steps taken to address this:

The manuscript initially incorrectly stated a 50% fistula incidence in the poor perfusion group. This was a typographical error; our original calculations (and the cited reference) used **65%**, derived from Chen et al. (Ref 31), who reported a 67% incidence of crPOPF in hypoperfused patients. We have corrected this value in the revised manuscript.

Our sample size calculation assumes a **two-sample proportion test with unequal group sizes**, using the normal approximation method. While exact tests (e.g., Fisher’s) often require larger samples, this approach is widely accepted for study design when preliminary effect sizes are based on prior data. The formula accounts for:

- Expected proportions (20% vs. 65%)
- A 4:1 enrollment ratio (clinically justified by the higher prevalence of normal perfusion cases)
- 80% power and a two-sided α = 0.05.

**Validation of Calculations:**
Using the script below (executed in R 4.3.3), we calculated a required **53 participants (42 normal perfusion, 11 poor perfusion)**. This aligns with the “pwr.2p2n.test” function using the “pwr” package when accounting for unequal group sizes, yielding 80.3% power. Our calculation provides a conservative yet feasible estimate for protocol planning.

- **R Script starts**

> # Load necessary package

> if (!require("pwr")) install.packages("pwr", dependencies = TRUE)

> library(pwr)

>

> # Define parameters

> p1 <- 0.20 # Proportion of fistulas in the normal perfusion group

> p2 <- 0.65 # Proportion of fistulas in the poor perfusion group

> alpha <- 0.05 # Significance level

> power <- 0.80 # Desired power

> ratio <- 4 # Imbalance ratio (n1/n2)

>

> # Calculate effect size for proportions

> effect_size <- ES.h(p1, p2)

>

> # Calculate pooled variance

> pooled_variance <- (p1 * (1 - p1) / ratio) + (p2 * (1 - p2))

>

> # Calculate required sample size for the smaller group (n2)

> n2 <- ((qnorm(1 - alpha / 2) + qnorm(power))^2 * pooled_variance) / (p1 - p2)^2

>

> # Scale for the larger group (n1)

> n1 <- n2 * ratio

>

> # Print results

> cat("Sample size for normal perfusion group (n1):", ceiling(n1), "\n")

Sample size for normal perfusion group (n1): 42

> cat("Sample size for poor perfusion group (n2):", ceiling(n2), "\n")

Sample size for poor perfusion group (n2): 11

> cat("Total sample size:", ceiling(n1 + n2), "\n")

Total sample size: 52

- **R Script ends**

Thank you again for your constructive feedback, which has strengthened our protocol’s rigor. Please let us know if further clarification would be helpful.

Sincerely,

The Authors
